# A tissue engineered 3D printed calcium alkali phosphate bioceramic bone graft enables vascularization and regeneration of critical-size discontinuity bony defects *in vivo*

**DOI:** 10.3389/fbioe.2023.1221314

**Published:** 2023-06-15

**Authors:** Christine Knabe, Michael Stiller, Marian Kampschulte, Janka Wilbig, Barbara Peleska, Jens Günster, Renate Gildenhaar, Georg Berger, Alexander Rack, Ulf Linow, Max Heiland, Carsten Rendenbach, Steffen Koerdt, Claudius Steffen, Alireza Houshmand, Li Xiang-Tischhauser, Doaa Adel-Khattab

**Affiliations:** ^1^ Department of Experimental Orofacial Medicine, Philipps University Marburg, Marburg, Germany; ^2^ Department of Prosthodontics, Philipps University Marburg, Marburg, Germany; ^3^ Department of Radiology, Justus Liebig University Giessen, Giessen, Germany; ^4^ Department of Biomaterials and Multimodal Processing, Federal Institute for Materials Research and Testing, Berlin, Germany; ^5^ Structure of Materials Group, ESRF (European Synchroton Radiation Facility), Grenoble, France; ^6^ Department of Oral and Maxillofacial Surgery, Charité University Medical Center Berlin (Charité-Universitätsmedizin Berlin), Corporate Member of Freie Universität Berlin, Humboldt-Universität zu Berlin, and Berlin Institute of Health, Berlin, Germany; ^7^ Department of Periodontology, Ain Shams University, Cairo, Egypt

**Keywords:** bioactive ceramics, 3D printed scaffold, bone tissue engineering, bone repair, calcium alkali orthophosphates, angiogenesis, angio-μCT, segmental discontinuity bone defects

## Abstract

**Introduction:** Recently, efforts towards the development of patient-specific 3D printed scaffolds for bone tissue engineering from bioactive ceramics have continuously intensified. For reconstruction of segmental defects after subtotal mandibulectomy a suitable tissue engineered bioceramic bone graft needs to be endowed with homogenously distributed osteoblasts in order to mimic the advantageous features of vascularized autologous fibula grafts, which represent the standard of care, contain osteogenic cells and are transplanted with the respective blood vessel. Consequently, inducing vascularization early on is pivotal for bone tissue engineering. The current study explored an advanced bone tissue engineering approach combining an advanced 3D printing technique for bioactive resorbable ceramic scaffolds with a perfusion cell culture technique for pre-colonization with mesenchymal stem cells, and with an intrinsic angiogenesis technique for regenerating critical size, segmental discontinuity defects *in vivo* applying a rat model. To this end, the effect of differing Si-CAOP (silica containing calcium alkali orthophosphate) scaffold microarchitecture arising from 3D powder bed printing (RP) or the Schwarzwalder Somers (SSM) replica fabrication technique on vascularization and bone regeneration was analyzed *in vivo*. In 80 rats 6-mm segmental discontinuity defects were created in the left femur.

**Methods:** Embryonic mesenchymal stem cells were cultured on RP and SSM scaffolds for 7d under perfusion to create Si-CAOP grafts with terminally differentiated osteoblasts and mineralizing bone matrix. These scaffolds were implanted into the segmental defects in combination with an arteriovenous bundle (AVB). Native scaffolds without cells or AVB served as controls. After 3 and 6 months, femurs were processed for angio-µCT or hard tissue histology, histomorphometric and immunohistochemical analysis of angiogenic and osteogenic marker expression.

**Results:** At 3 and 6 months, defects reconstructed with RP scaffolds, cells and AVB displayed a statistically significant higher bone area fraction, blood vessel volume%, blood vessel surface/volume, blood vessel thickness, density and linear density than defects treated with the other scaffold configurations.

**Discussion:** Taken together, this study demonstrated that the AVB technique is well suited for inducing adequate vascularization of the tissue engineered scaffold graft in segmental defects after 3 and 6 months, and that our tissue engineering approach employing 3D powder bed printed scaffolds facilitated segmental defect repair.

## Introduction

Due to the significant advances in the areas of additive manufacturing technologies for ceramics and tissue engineering there have been ever increasing efforts to develop patient-specific bioceramic scaffolds (Adel-Khattab et al., 2018) and adequate vascularization approaches for bone tissue engineering for regeneration of large segmental discontinuity defects.

**FIGURE 1:**
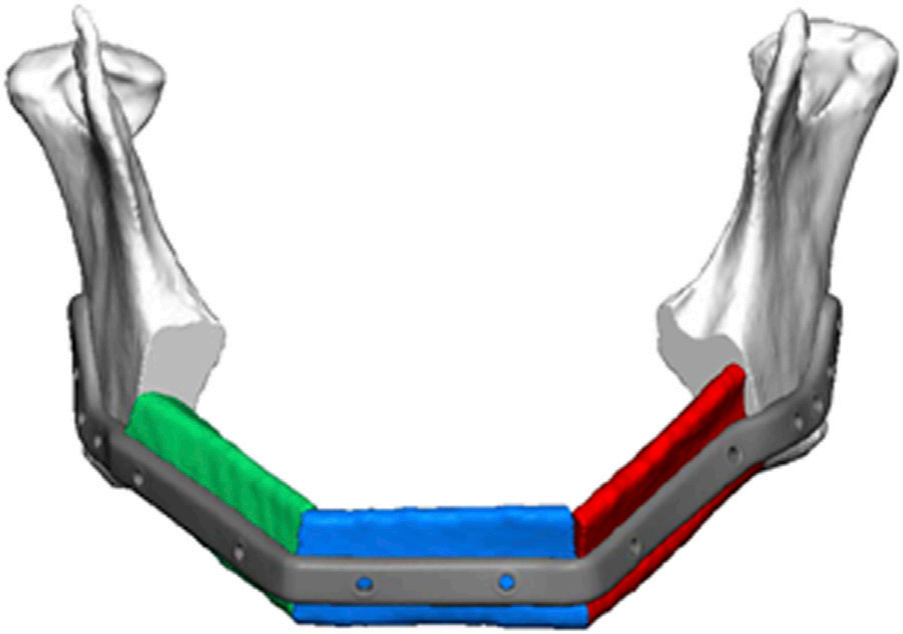
Schematic of segmental mandibular defect extending for more than 10 cm from ascending ramus to ascending ramus as a result of tumor ablation. Placement of a patient specific reconstruction plate in combination with vascularized fibular grafts (depicted in red, green, and blue) is anticipated, which represents the clinical standard of care. The regular wound healing process does not allow cells to migrate from the defect margins over a distance of this size to colonize large segmental defects more than 5 cm in size or scaffolds placed into these defects. Consequently, osteogenic cell containing bone grafts of scaffolds endowed with osteogenic cells need to be used in combination with an adequate vascularization approach for reconstruction of these type of defects (*Compliments of Professor C. Rendenbach and KLS Martin, GmbH, Germany*).

Reconstruction of extensive and complex bone segments resulting from tumor surgery involving subtotal mandibulectomy (Figure 1) constitutes a challenging problem in craniomaxillofacial surgery (Warnke et al., 2004; Cancedda et al., 2007; Eweida et al., 2012), and often necessitates reconstructing defects, which extend over a length of more than 10 cm from ascending ramus to ascending ramus of the mandible (Warnke et al., 2004; Lonie et al., 2016; Kreutzer et al., 2022; Ruf et al., 2022) (Figure 1). In this context, clinical principles demand, that, when reconstructing segmental mandibular defects 5 cm in width or larger, a vascularized bone graft needs to be used, which contains osteogenic cells and is transplanted with the respective blood vessel, which is then anastomosed with a blood vessel of the recipient site using microvascular surgery. This is due to the fact that the regular wound healing process does not allow cells to migrate from the defect margins over too large a distance to colonize these large segmental defects or scaffolds placed into these defects. The same is true for timely ingrowth of blood vessels from the defect margins. As a result, the clinical standard of care for bone reconstruction after subtotal mandibulectomy involves the use of vascularized fibular (Figure 1) or iliac crest grafts, which contain both osteogenic cells and a blood vessel (Eweida et al., 2014; Lonie et al., 2016; Kreutzer et al., 2022; Ruf et al., 2022; Warnke et al., 2004.). However, in addition to the significant risk of donor-site morbidity, the use of vascularized fibular grafts does often not permit an exact reconstruction of the patient’s original anatomic facial and mandibular features, thereby leading to a compromised esthetic and functional result, which also affects the outcome achieved when fabricating the implant supported dental prosthesis. As a result, there has been an increasing search for adequate tissue engineering approaches and scaffolds, which, when reconstructing defects after subtotal mandibulectomy, need to be endowed with osteogenic cells in addition to ensuring adequate vascularization in order to provide these cells with oxygen and nutrients (Warnke et al., 2004; Cancedda et al., 2007; Eweida et al., 2012; Eweida et al., 2014; Eweida et al., 2015; Arkudas et al., 2015; Laschke and Menger, 2016). An ideal scaffold should be resorbable and bioactive, i.e., have a stimulatory effect on bone tissue formation, and be remodeled into functional bone tissue resulting in restoration of the original bone microarchitecture.

Whereas first bone tissue engineering approaches involving bioceramics, mesenchymal stem cells and vascularization techniques with and without the addition of bone morphogenetic proteins (BMPs) mostly employed bioceramic granules (Warnke et al., 2004; Eweida et al., 2014; Horch et al., 2014), over the last decade, significant progress has been made with adapting powder bed binder jet (ink jet) 3D printing, robocasting, selective laser sintering, stereolithography, and layer-wise slurry deposition binder jet 3D printing technologies so as to enable 3D printing of bioceramic scaffolds (Gildenhaar et al., 2012; Travitzky et al., 2014; Shirazi et al., 2015; Zanchetta et al., 2015; Zocca et al., 2015; Ishack et al.,. 2017; Zocca et al., 2017; Adel-Khattab et al., 2018). These recent advances in 3D printing techniques for bioceramics include advances with respect to increased printing resolution and enhanced control over porosity, strut size and thereby scaffold microarchitecture (Tarafder et al., 2013; Tian et al., 2013; Shirazi et al., 2015; Zocca et al., 2015; Zocca et al., 2017; Adel-Khattab et al., 2018). They furthermore open the possibility to fabricate patient-specific bioactive resorbable implants and scaffolds which possess exactly the same morphology as the patient defect (Adel-Khattab et al., 2018).

Various synthetic bioactive calcium phosphate-based bioceramic bone substitute materials have been proposed for use in bone tissue engineering such as bioactive glass 45S5 (BG45S5) and tricalcium phosphate (TCP) (Yao et al., 2005; Handel et al., 2013; Bose et al., 2017). These also include the more recently developed silica containing calcium alkali orthophosphate (Si-CAOP), which has been developed for higher bioactivity and resorbability than TCP and BG45S5, which have been shown to exhibit excellent osteoconductivity and resorb within 1–2 years in the human case (Tadjoedin et al., 2002; Zerbo et al., 2001; Zerbo et al., 2004; Zerbo et al., 2005; Zijderveld et al., 2005; Aghaloo and Moy, 2007; Pjetursson et al., 2008; Zijderveld et al., 2009; Knabe et al., 2008a; Knabe et al., 2017b; Knabe et al., 2017c). Si-CAOP has previously been shown to display a greater enhancement effect on osteogenic cell differentiation *in vitro* and bone formation *in vivo* than TCP, both in clinically representative large animal models as well as in patients. This was in combination with a favorable biodegradability (Knabe et al., 2004; Knabe et al., 2005; Knabe and Ducheyne, 2008; Knabe et al., 2008b; Knabe et al., 2017a; Knabe et al., 2017d; Knabe et al., 2017e; Adel-Khattab et al., 2018). The stimulatory effect on osteogenesis *in vitro* and *in vivo* was associated with simultaneous enhanced activation of the ERK differentiation, P3IK cell survival and alternate p38 intracellular signaling pathways (Knabe et al., 2017d). This was in addition to enhanced fibronectin adsorption and cell adhesion being mediated predominantly via the α_5_β_1_ fibronectin receptor and to a lesser degree via the α_2_β_1_ collagen receptor (Knabe et al., 2017d). Collectively, these findings rendered Si-CAOP a promising bone grafting material for developing binder jet 3D powder bed printed bioceramic scaffolds (Adel-Khattab et al., 2018). In addition to the chemical composition of the bioactive scaffold ceramic material, which greatly affects bone formation and repair, also the macro- and microarchitecture of the scaffolds have a major effect on bone ingrowth, regeneration, and on vascularization (Mastrogiacomo et al., 2006). In this context, different manufacturing technologies render 3D bioceramic scaffolds with considerable differences in microarchitecture such as macro- and microporosity, pore size distribution and interconnected porosity (Travitzky et al., 2014; Zocca et al., 2015; Zocca et al., 2017), which have been shown to affect the pattern of blood vessels invasion, the kinetics of the bone neoformation process, and the amount of bone deposition (Mastrogiacomo et al., 2006), even when using the same material.

In the present study, 3D Si-CAOP scaffolds manufactured by 3D binder jetting powder bed printing were compared to SSM scaffolds fabricated by the conventional SSM replica technique, which produces scaffolds which resemble the microarchitecture of natural cancellous bone. 3D printing on the other hand possesses greater control with respect to reproducibility and enables fabricating scaffolds which display a more uniform pore size, architecture and interconnecting porosity (Bose et al., 2017).

Utilizing 3D printed Si-CAOP scaffolds in combination with perfusion flow culture of mesenchymal stem cells, we previously were able to create a 3D printed tissue engineered synthetic bone graft *in vitro*, with homogenously distributed osteoblasts and mineralizing bone matrix (Adel-Khattab et al., 2018), which mimics the favorable features of autologous bone grafts thereby making it an excellent candidate for subsequent *in vivo* implantation for repair of extensive segmental discontinuity bony defects such as after subtotal mandibulectomy (Warnke et al., 2004; Lonie et al., 2016; Ruf et al., 2022).

However, obtaining sufficient vascularization of tissue engineered constructs particularly in the center of large 3D scaffolds, which have been pre-cultured with osteogenic stem cells, is of paramount importance (Cancedda et al., 2007; Kirkpatrick et al., 2011), since the reason for failure of classical bone tissue engineering strategies has most often been the lack of vascularization within the tissue engineered bone constructs, leading to poor tissue integration and implant survival (Cancedda et al., 2007; Eweida et al., 2012; Eweida et al., 2014; Eweida et al., 2015). The maximum distance by which cells can be separated from the nearest blood vessel with respect to maintaining a sufficient supply of oxygen and nutrients by diffusion to ensure their survival, is 150–200 μm (Yu et al., 2009; Cai et al., 2011). Consequently, inducing angiogenesis and achieving vascularization early on during wound healing is crucial for bone tissue engineering. Efficient vascularization is not only important for cell survival but also for growth factor and cytokine production by mesenchymal stem cells, which promotes bone metabolism and repair and thus effective integration of tissue engineered grafts after *in vivo* implantation. In this context, it is noteworthy, that osteoblasts and endothelial cells act as drug delivery system for each other by secreting important growth factors and thereby mutually enhance each other’s cell function (Kirkpatrick et al., 2011). In addition, abundant blood supply prevents infection (Han et al., 2014).

Most currently applied regenerative approaches rely on extrinsic vascularization with the neovascular bed originating from the tissue bed outside of the defect rather than being formed within the grafted defect so as to enable adequate perfusion of extensive grafts. The intrinsic vascularization concept on the other hand is founded on the principle that an artery or vein serve as sources of new capillary formation within the grafted or newly formed tissue. To this end, the “intrinsic angiogenesis chamber” technique entails the creation of an arteriovenous loop (AVL) (Lokmic et al., 2007; Lokmic and Mitchell, 2008a; Lokmic and Mitchell, 2008b; Eweida et al., 2014; Fan et al., 2014) or an arteriovenous bundle (AVB) (Tanaka et al., 2003; Houben et al., 2021; Ismail et al., 2021) utilizing microvascular surgical techniques. The AVL technique is based on the discovery of Erol and Spira (1980), who showed that by creating an AV loop spontaneous sprouting of blood vessels out of this loop was achieved. The AVL serves as a source for neoangiogenesis and formation of a network of capillaries beginning to form at the 3rd postoperative day with angiogenesis reaching its peak at day 10 (Lokmic et al., 2007; Lokmic and Mitchell, 2008a), resulting in a robust vascular network (Mian et al., 2000; Messina et al., 2005; Morritt et al., 2007). Tanaka et al. (2003) found the AVB technique using a femoral AVB to be an effective and practical vascular carrier for producing a tissue-engineered skin flap. Taken together, the intrinsic vascularization approach has been found to result in a considerably greater angiogenic and osteogenic effect than extrinsic vascularization (Kokemueller et al., 2010; Fan et al., 2014). Over the last decade, the AVL and AVB approaches have been further adapted for their use in tissue engineering (Ren et al., 2008; Rath et al., 2012; Ismail et al., 2021). The AVL approach was utilized in sheep and goats to achieve vascularization of a growth chambers filled with *β*-TCP-hydroxyapatite (HA) granules in combination with autogenous mesenchymal stem cells (MSCs) and rhBMP-2 (bone morphogenetic protein-2) or in a goat model for the reconstruction of critical-size (but not segmental) mandibular defects using a scaffold composed of 60% HA/40% TCP in combination with autogenous platelet-rich plasma (PRP) and BMP-2 (Eweida et al., 2014). Furthermore, Horch et al. (2014) published the first two patient cases, in which the AVL technique was successfully used for bone repair of defects in the radius and ulna by combining *β*-TCP/HA, fibrin glue and bone marrow aspirate from the iliac crest with an AVL. More recently, Ismail et al., 2021 published a patient case, in which an AVB was used to pedicle an ectopically created tissue engineered bone graft, which was created by combining an allogenic devitalized bone matrix, stromal cells and BMP-2, which were wrapped in the latissimus dorsi muscle and then used to reconstruct a complex hemimaxillary defect, for which the AVB was anastomosed with the vasculature of the recipient site using microvascular surgery. This approach succeeded in reconstructing the orbital floor, in achieving separation of the oral and nasal cavities, and midface symmetry. It furthermore allowed the patient to return to normal diet as well as to restore normal speech and swallowing function. Consequently, the intrinsic angiogenesis approach utilizing AVLs of AVBs appears to be a promising strategy for vascularizing bone tissue engineered constructs.

The objective of this study was to explore an advanced bone tissue engineering approach combining an advanced binder jet 3D powder bed printing technique for bioactive resorbable ceramic scaffolds with an advanced perfusion flow cell culture technique for pre-colonization of these scaffolds with mesenchymal stem cells, and with an intrinsic AVB angiogenesis technique for regenerating critical size, segmental discontinuity defects *in vivo* applying rat model. Consequently, the specific aims of this study entailed evaluating the effect of 3D printed bioactive Si-CAOP scaffolds, which were endowed with homogenously distributed osteoblasts and mineralizing bone matrix as a result of *in vitro* pre-colonizaton, on angiogenesis, bone formation and segmental bone defect repair *in vivo* when using a microvascular AVB technique as compared to that of Si-CAOP scaffolds produced using the Schwarzwalder Somers (SSM) replica technique. This included analyzing the effect of differing scaffold microarchitecture arising from 3D powder bed printing (i.e., rapid prototyping, RP) or the Schwarzwalder Somers (SSM) replica fabrication technique on vascularization and bone regeneration employing a well-established femoral segmental bone defect model in the rat (Oest et al., 2007).This was, furthermore, to achieve proof of concept prior to advancing with the most suitable scaffold configuration to a large animal mini-pig model featuring a critical size segmental mandibular defect following well established translational and animal ethics principles (Supplementary Figure S1).

## Materials and methods

### Preparation of bioceramic scaffolds

#### Scaffold base bioceramic material

Scaffolds were prepared from a silica containing CAOP powder (Si-CAOP, code GB9S14) whose composition and synthesis have been described in detail elsewhere (Adel-Khattab et al., 2018). In brief, Si-CAOP features the main crystalline phase Ca_2_KNa(PO_4_)_2_ and (Schneider et al., 1994) a small amorphous portion containing MgKPO_4_ and a 4 wt% SiO_2_ glass phase, which served as sintering aid. The GB9S14 Si-CAOP composition was as follows: main component (96%) - CaO (30.67 wt%), P_2_O_5_ (43.14wt%), Na_2_O (9.42wt%), K_2_O (14.32wt%), MgO (2.45wt%) to which the 4% SiO_2_ sintering aid was added. The main component was fabricated by planetary ball milling of a melt resulting from mixing Ca_2_CO_3_, MgO, Na_2_CO_3_, K_2_CO_3_ and H_3_PO_4_ followed by milling, calcination and heat treatment at 1,600°C.

#### Fabrication of SSM scaffolds

Cylindrical SSM Scaffolds 6 mm in length and 5 mm in diameter were fabricated utilizing the SSM replica technique as depicted and described in detail previously (Adel-Khattab et al., 2018). In brief, a polyurethane sponge template featuring irregular interconnecting pores (60 pores per inch (60 ppi), size 200–300 µm) was coated with a slurry prepared from GB9S14 powder (mean grain size: 12.7 µm), followed by pyrolysis of the polymer and sintering, which resulted in bioceramic scaffolds displaying the microarchitecture of the sponge (Adel-Khattab et al., 2018).

#### Fabrication of RP scaffolds

For the fabrication of 3D printed cylindrical Si-CAOP scaffolds 6 mm in height and 5 mm in diameter binder-jet 3D powder bed printing was utilized employing an ExOne™ 3D printer of the R1Series (ExOne LLC, North Huntingdon, PA, United States), as described and depicted in detail elsewhere (Adel-Khattab et al., 2018). In short, Solid Edge CAD-software was used to generate the STL file for the 3D printing of scaffolds which displayed channels that passed through the scaffolds both horizontally and vertically. For the binder jet 3D powder bed printing process Si-CAOP powder with a grain size of 45–90 µm was generated employing fluidized bed granulation. Binder jet 3D printing was carried out at a resolution of 100 µm employing an aqueous binder and a printing head with a nozzle size of 63 µm. The 3D printing process was followed by depowdering, drying and sintering, as described in more detail previously (Adel-Khattab et al., 2018).

#### Scaffold material properties

The material properties such as porosity and pore size distribution, dissolution behavior, selective ion release, surface area and compressive strength of both scaffold types have been characterized in depth in a previous study (Adel-Khattab et al., 2018). In brief, while SSM scaffolds exhibited a considerably greater overall porosity of 86.9% when compared to 3D printed RP scaffolds, which had an overall porosity of 50%, RP scaffolds displayed a significantly greater microporosity in combination with a higher percentage of pores ≥600 µm in size. In detail, the distribution of the sizes of the open pores was for SSM scaffolds 8.8% of the pores ≥600 µm, 84.2% of pores 200–600 μm, 3.8% of pores 50–200 μm, and 3.2% of pores ≤50 μm; and for 3D printed RP scaffolds: 32.8% of pores ≥600 µm, 18.5% pores 200–600 μm, 10.7% of pores 50–200 μm, and 38% of pores ≤50 µm. Furthermore, SSM scaffolds exhibited a surface area of 0.081 m^2^/g and a compressive strength of 0.46 ± 0.2 MPa; and RP scaffolds a surface area of 0.047 m^2^/g and a compressive strength of 6.6 ± 0.8 MPa. With respect to dissolution behavior, while SSM scaffolds exhibited a considerably higher total ion release, selective ion release for silica, magnesium- and calcium-ions was significantly higher with RP scaffolds. In detail, SSM scaffolds displayed a total ion release of 180 mg/g after 70 days of immersion in TRIS-HCl solution (20 mL per 1 g powder, 37°C, pH 7.4) and RP scaffolds a total ion release of 120 mg/g. In contrast, selective ion-release for silica was 0.07 mg/g for SSM scaffolds vs. 2.59 mg/g for RP scaffolds; for calcium 0.16 mg/g with SSM scaffolds vs. 0.55 mg/g with RP scaffolds; for magnesium 5.3 mg/g for SSM scaffolds and 3.6 mg/g for RP scaffolds; and for phosphate-ions 25.5 mg/g with SSM scaffolds and 14.5 mg/g with RP scaffolds.

### Presurgical colonization of scaffolds with osteogenic stem cells under dynamic cell culture conditions

Primary embryonic mesenchymal rat stem cells (MSCs), which were obtained from embryonic rat calvaria (R-OST-583 Lonza, Switzerland) and which were committed to the osteoblastic lineage by utilizing an adequate osteogenic culture medium, were seeded and cultured dynamically for 7 days on 3D printed RP and on SSM scaffolds of group 1 and 2, as outlined in detail previously (Adel-Khattab et al., 2018). These embryonic rat mesenchymal stem cells (MSCs) were committed to the osteoblastic lineage by using an osteogenic culture medium with the following composition: Dulbecco’s Modification of Eagle’s Medium without L-Glutamine, with 4.5 g/L Glucose and Sodium Pyruvate (Gibco, Paisley, UK) containing 10% fetal calf serum and antibiotics (30 μg/mL gentamicin, 15 pg/mL amphotericin B) supplemented with 2 mM L-glutamine, 50 mg/mL ascorbic acid, 5 mmol glycerophosphate (Sigma, St. Louis, MO, United States) and 10^–8^ M dexamethasone. Cells were expanded in a 37°C, humidified, 5% CO,/95% air incubator. For the *in vivo* experiments in the rat animal model cell seeding of these osteogenic rat stem cells was carried out at a concentration 3 × 10^6^ cells per ml under dynamic flow conditions of 0.2 mL/min on both SSM and RP scaffolds (group 1 and 2) for 24 h. Subsequently, the circuit configuration was changed as described previously (Adel-Khattab et al., 2018), and dynamic perfusion flow conditions of 0.5 mL/min were used for culturing the cells on both types of scaffolds for 7 days prior to *in vivo* implantation. These specific seeding and culture condition were selected based on a previous study, which showed that these experimental conditions reliably facilitated a homogenous distribution of terminally differentiated osteoblasts and mineralizing bone matrix throughout both types of scaffolds after 7 days of dynamic culture thereby creating a Si-CAOP bone graft *in vitro*, which mimicked the advantageous features of autologous bone grafts in that it was endowed with terminally differentiated osteoblasts and mineralizing bone matrix at the time of *in vivo* implantation (Adel-Khattab et al., 2018). This is in addition to permitting a manufacturing process which can fabricate synthetic bone grafts that exactly match the morphology of the patient defect based on the patient-specific CT data (Adel-Khattab et al., 2018).

### *In vivo* rat study

#### Experimental design *in vivo* rat study

In the current study, a femoral segmental defect model was used in 80 six-month old female Wistar rats involving creation of a 6-mm long critical size segmental defect in the left femur of each animal (Oest et al., 2007). In 20 rats a 3D printed RP (rapid prototyping) scaffold, on which MSCs had been cultured dynamically for 7 days, was implanted in this segmental discontinuity defect in combination with an AVB (group 1, denominated: RP, cells & AVB) for 3 (group 1a, *n* = 10) and 6 months (group 1b *n* = 10). Another 20 rats received an SSM scaffold, on which rat MSCs had been cultured dynamically for 7 days, in combination with an AVB (group 2, denominated: SSM, cells & AVB) for 3 (group 2a, *n* = 10) and 6 months (group 2b, *n* = 10). Native 3D printed RP and SSM scaffolds (without use of cells or the AVB technique) served as controls. Thus, the remaining rats received either a RP scaffold (group 3, denominated: RP control) or a SSM scaffold (group 4, denominated: SSM control) for 3 and 6 months (RP scaffold, 3 months, group 3a, *n* = 10; RP scaffolds 6 months, group 3b, n = 10), SSM scaffolds 3 months, group 4a, *n* = 10; SSM scaffolds 6 months, group 4b, *n* = 10. Within each group 5 animals were used for angio-micro-computed tomographical (angio-µCT) analysis, which required decalcification of the bone samples, and 5 animals were used for undecalcified hard tissue histology, histomorphometric and immunohistochemical analysis.

#### Surgical procedures

**FIGURE 2:**
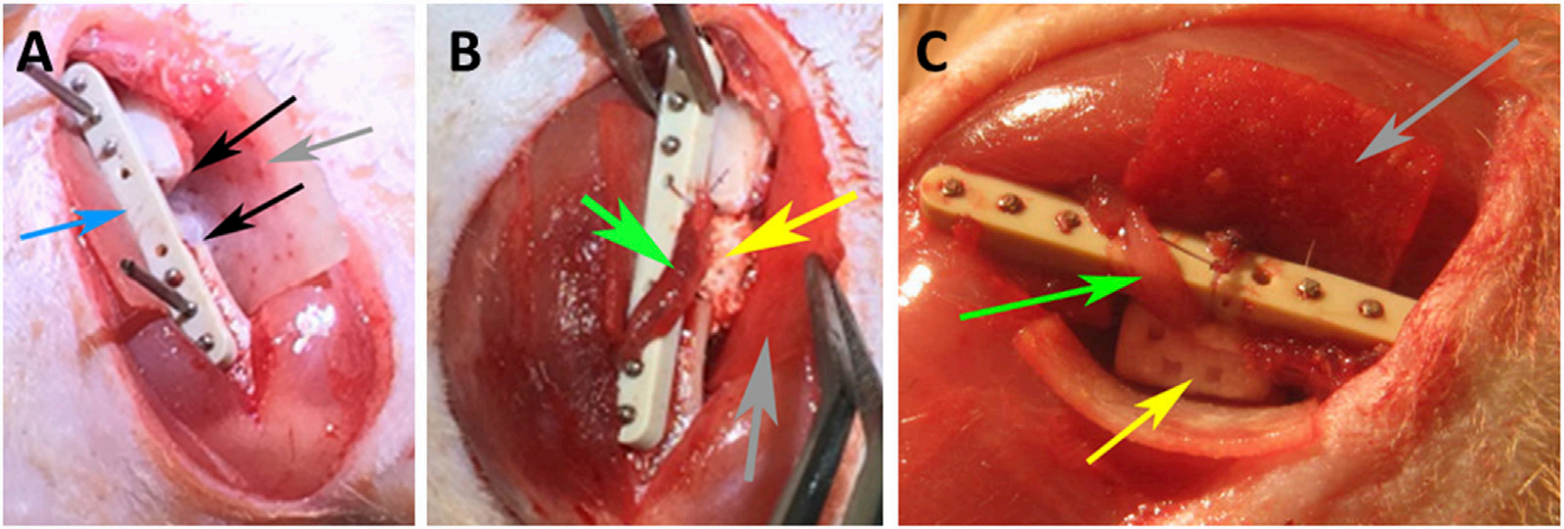
Intraoperative photographs of the rat surgeries, **(A)** depicting the critical size segmental defect (black arrows), osteosynthesis plate–blue arrow; **(B)** implantation of a SSM scaffold (yellow arrow); **(C)** placement of a 3D printed RP scaffold (yellow arrow); arteriovenous bundle (AVB)—green arrow, collagen membrane–grey arrow.

The design of the animal experimentation was officially reviewed, and animal ethics approval was granted by the local authorities (reference No. V 54–19 c 20 15 h 01 MR 20/25) in accordance with the German and European Animal Welfare guidelines. All surgical procedures were carried out under general anesthesia by intraperitoneal injection of 75 mg/kg ketamine, 5–8 mg/kg Xylacin, 0.2 mL/100 g weight of the rat with administration of tramadol (20 mg/kg) for analgesia also via intraperitoneal injection during general anesthesia of the animals, which on the average were 354 g in weight. First, the left lower extremity of the rats was extended, shaved and fixed with patches to the operating table. After sterile covering of the surgical field, a transverse incision was performed in the course of the inguinal ligament. A critical size defect was created in all groups; and an AVB was prepared and positioned in the center of the defects/scaffolds in group 1 and 2. The AVB was prepared from the same side of the femur, by preparing the femoral artery and vein as a bundle using a microsurgical technique. Placement of an osteosynthesis plate (RIS system, Davos, Switzerland) utilizing 6 screws was followed by creating a 6-mm long critical size segmental bony discontinuity defect in the left femur with a Gigly wire saw, while the osteosynthesis plate and the six fixation screws facilitated defect stabilization (Le et al., 2001) (Figures 2A–C). One scaffold of the four experimental scaffold groups (per animal) was inserted press fit into these defects, and finally covered by a collagen membrane (Collprotect^®^ (Botiss Inc., Berlin, Germany). In group 1 and 2 the AVB was guided through the muscle bundles, transposed directly to the center of the scaffold and sutured to the collagen membrane so as to prevent any dislocation utilizing a 6–0 resorbable suture (SERAFAST^®^ Undyed, SERAG-WIESSNER) (Figures 1B, C). Multilayer suturing for wound closure involved utilizing horizontal mattress and interrupted 3–0 resorbable sutures (VICRYL plus Ethicon Polyglactin 910). Skin suturing was performed by using interrupted 5–0 non resorbable sutures (Monocryl Ethicon Sutures 5/0 P-3 13 mm 45 cm Undyed Y493G). In the first 6 postoperative days 20 mg/kg of tramadol (Tramadol-Mepha^®^ (CH)) were administered in the drinking water for postoperative pain relief and 50 mg/mL of Enrofloxacin (BaytrilWDT, WDT, Germany) for antibiotic prophylaxis. In cases of severe pain 4 mg/kg of Rimadyl were given up to 24 h. After 7 days, the initial wound healing was assessed, checked and sutures were removed.

#### Euthanasia and sample collection

At two time points three and 6 months postsurgically euthanasia of the rats was performed under general anesthesia by inhalation of CO_2_. For angio-µCT analysis perfusion with a contrast agent was performed as described by Kampschulte et al. (2016). In brief, puncturing of the abdominal aorta was followed by cannulation with a 24 G i.v. Catheter and injection of 10 mL of a 0.9% NaCl-solution for achieving effluent free of blood. Subsequently, injection of 10 mL of a lead containing silicone polymer (Microfil^®^ MV-122, Flow Tech, Carver, MA, United States) was carried out for perfusion with the contrast agent, which was allowed to polymerize for 60 min. Then the femora were removed, fixed in formalin and decalcified in 5% EDTA/0.1 M Tris–HCL, pH 7.3, before μCT acquisition was performed. Decalcification of the bone samples was required because of the similar radiographic densities of the bioceramic scaffolds, bone and the contrast agent.

#### Angio-micro-computed tomography analysis

**FIGURE 3:**
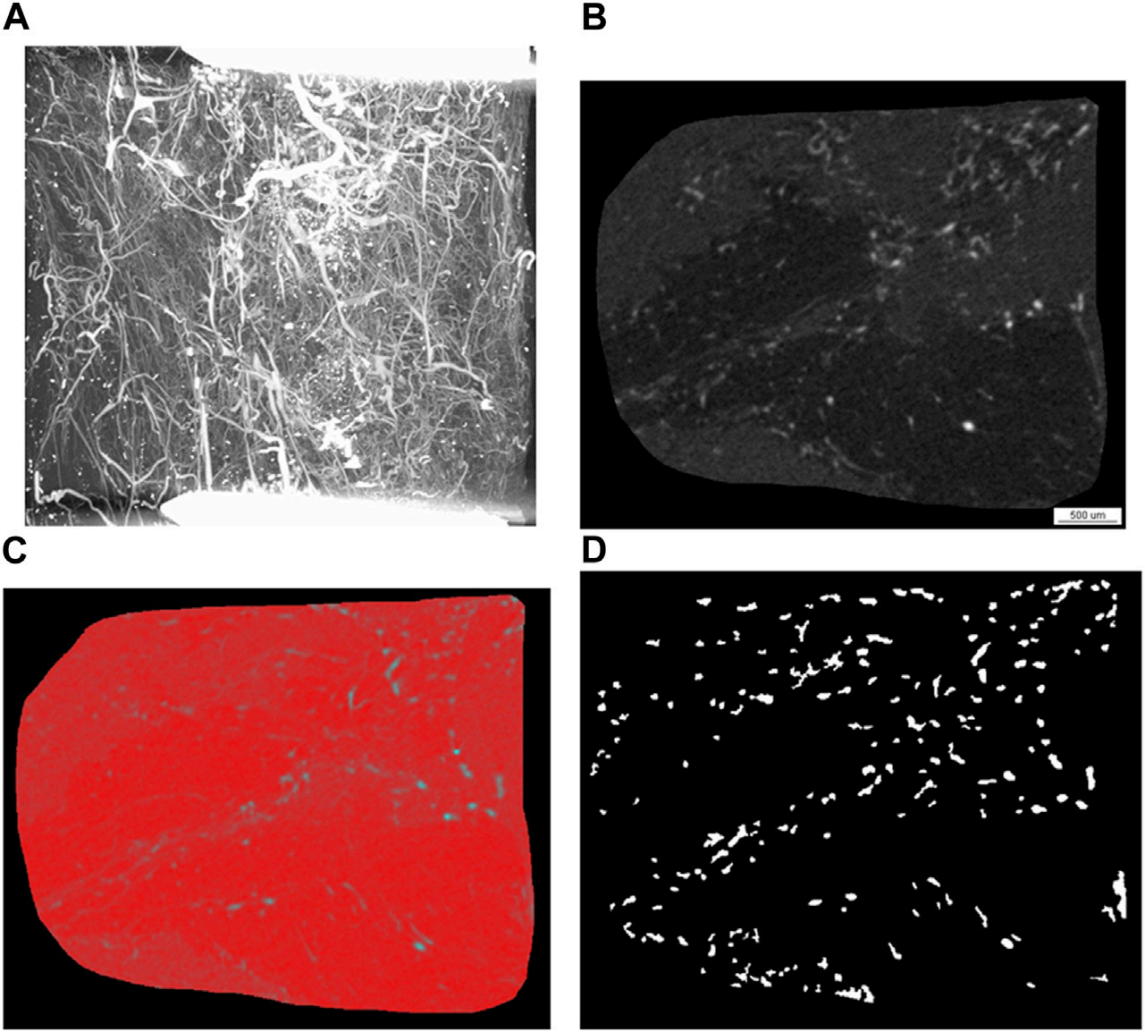
Representative images of the angio-µCT analysis. Angio-µCT analysis involves image and raw data acquisition and analysis of a stack of 857 slices, which entails grey scale transformation **(B)**, smoothing and noise reduction, segmentation **(C)**, segmentation of foreground from background, operations based on binary arithmetic, binarization, removal of speckles from images and calculation of 3D parameters from binary images **(D)** employing custom made algorithms of the CTAn CT analyzer software as well as a sphere fitting algorithm for determining thickness. **(A)** Angio-µCT image (maximum intensity projection), which visualizes the vasculature within the segmental critical size defect, the fixation screws of the osteosynthesis plate, which are located outside of the defect appear as white bars, decalcification allows a depiction of the microvessels in the critical size defect; **(B)** grey scale image generated after grey scale transformation, **(C)** grey-scale image with red false-color overlay of binary segmentation **(D)** binary image of the region of interest, in which radiopaque dots correspond to blood vessels. The 3D parameters are calculated from these binary images employing custom made algorithms of the CTAn CT analyzer software as well as a sphere fitting algorithm for determining thickness. Taken together the following parameters are calculated: blood vessel volume fraction, blood vessel surface/blood vessel volume, blood vessel thickness, blood vessel density and blood vessel linear density.

A high energy micro-computed tomography (microCT) system (µCT 1,173, Bruker, Kontich, Belgium) was utilized for scanning the entire decalcified femora with the following setting: spatial resolution- 7.1 μm isotropic voxel side length, no filter, 4-fold frame averaging, 0.2° rotation steps. The tube voltage was - 130 kVp, and the tube current - 60 μA. A Feldkamp algorithm was applied for generating images with an 8-bit gray-scale resolution from the projection data (Figure 3). CTAn CT analyzer software (Bruker, Kontich, Belgium) was employed for carrying out the various measurements and analyses. Image stacks for the defect area 6 mm in length were selected within the decalcified femora based on the distance between the two fixation screws adjacent to the defect margins. Acquisition of gray-scale image sets of the regions of interest (ROI) (Figure 3B) was followed by binarization employing a locally adaptive binarization technique in order to identify the vasculature (Figures 3C, D). Analysis of vascularity excluded areas with high-density gray values which represented metal particle artifacts originating from metal particles (Figure 3D). The contralateral untreated femora served as reference.

The following morphometry parameters were measured using the CTAn CT analyzer software (Bruker, Kontich, Belgium):

The vascular volume fraction (blood vessel volume %) was calculated using the following equation:

$$Vascular\;volume\;fraction\;VVF=\frac{3D\_TCAV}{3D\_TTV}*100\%$$

The sum of all pixels marked as contrast agent subsequent to thresholding yields the TCAV (total (contrast agent volume), TCAS (total contrast agent surface) denotes the surface of the binarized objects and TTV (total tissue volume) represents the sum of all pixels within the ROI (Kampschulte et al., 2016).

The vascular surface/vascular volume (blood vessel surface/blood vessel volume) was calculated using the following equation:

$$Vascular\;surface/vascular\;volume=\frac{3D\_TCAS}{3D\_TCAV}\left(1/mm\right)$$

Vascular (blood vessel) thickness (µm).

Vascular (blood vessel) density (1/mm). The blood vessel density represents the blood vessel area (mm^2^) per unit volume (mm^3^).

Vascular (blood vessel) linear density (1/mm). The blood vessel linear density was calculated as the total vascular length (mm) divided by the total area of the ROI (mm^2^).

A sphere fitting algorithm was employed for determining structure thickness as described by Hildebrand and Ruegsegger (1997).

#### Histomorphometric and immunohistochemical analysis for angiogenic and osteogenic markers

Five specimens per experimental group (group 1–4, i.e., RP and SSM scaffolds with and without cells and AVB), and per time point (three and 6 months) were subjected to undecalcified hard tissue histologic and immunohistochemical analysis without removing the bioceramic biomaterial from the tissue blocks and sections, which is the case with routine decalcified paraffin embedding and sections. An advanced resin embedding hard tissue histologic technique was employed, so as to facilitate examining the cell and tissue response at the bone-bioceramic material interface at a molecular level. For histologic processing, the ends of the femora were removed in order to obtain a femoral segment which contained the critical size 6-mm defect region and 3 mm of the adjacent native bone. This was followed by fixing the femoral bone blocks in an ethanol-based fixative (HistoCHOICE^R^, Amresco, United States) at RT (20°C–22°C) for 5 days, and processing, as described in detail elsewhere (Knabe et al., 2006; Knabe et al., 2008a; Knabe et al., 2017b; Knabe et al., 2017c). In brief, resin embedding was performed utilizing a mixture of methylmethacrylate and butyl-methacrylate and polymerizing conditions which maintained the antigenicity of the tissue. After gluing the polymerized blocks to acrylic slides (Plexiglas slides #404150/GLS, Walter Messner GmbH, Germany) 50 μm-thick sections were cut parallel to the long axis of the scaffolds and femora with a Leitz 1,600 sawing microtome (Leitz, Wetzlar, Germany) followed by grinding and polishing. The following primary antibodies were employed for immunohistochemical staining, which was described in detail previously (Knabe et al., 2017b; Knabe et al., 2017c), in order to analyze expression of osteogenic and angiogenic markers: monoclonal mouse anti-collagen type IV, anti-collagen type I, (Col I), anti-osteocalcin (OC), anti-bone sialoprotein (BSP), anti-alkaline phosphatase (ALP) antibodies (Abcam Cambridge, UK), and rabbit polyclonal antibodies against von Willebrand factor (vWF) (Abcam ab6994), and CD-31 (Abcam ab119339). Non immunized mouse, and rabbit IgG (PP54 and PP64) (Millipore, Billerica, Massachusetts, United States) served as negative controls to rule out non-specific reactions of mouse and rabbit IgG as outlined previously (Knabe et al., 2017b; Knabe et al., 2017c). Incubation with primary antibodies was followed by application of a peroxidase labelled dextran polymer conjugated to goat anti-mouse and anti-rabbit immunoglobulins and a 3-amino-9-ethylcarbazole (AEC) system for color development (DAKO, Glostrup, Denmark). Counterstaining was carried out with Mayer’s hematoxylin and mounting of coverslips with Kaiser’s glycerol gelatine (Merck KGaA, Darmstadt, Germany).

These longitudinal sections were subjected to histomorphometric analysis employing a light microscope (BX63; Olympus, Hamburg, Germany) and digital camera (DP73, Olympus, Hamburg, Germany) in combination with cellSens™ software. The following parameters were measured and analyzed as percentage of the total: the bone area fraction, the scaffold material area fraction for characterizing the biodegradability of the Si-CAOP scaffolds, and the bone-bioceramic contact for characterizing the bone bonding behavior of the scaffold bioceramic material. For determining the bone-bioceramic contact, first the total length of the bioceramic-tissue interface was measured in the undecalcified tissue sections using the cellSens™ software. This was followed by measuring the length of the bone-bioceramic contact. And the bone-bioceramic contact was then expressed as percentage of the total length. In addition, semi-quantitative analysis of expression of osteogenic and angiogenic markers in the various cell and matrix components of the tissue was carried out by two experienced investigators on the immunohistochemically stained sections as outlined in detail previously (Knabe et al., 2008a; Knabe et al., 2017b; Knabe et al., 2017c). For quantifying the amount of staining with respect to osteogenic marker expression, a semi-quantitative scoring system was applied which assigned a score of (**+++**[ = 5]), (**++**[ = 4]), and (**+**[ = 2]) to generalized strong, moderate or mild staining, and a score of (+++[ = 4]), (++[ = 3]), and (+[ = 1]) to strong, moderate or mild staining in localized regions. A score of (0) was given for no staining. The scores for the amount of staining for a respective osteogenic marker in a given cellular or matrix component were then averaged, and an average score of 3.5–5 was assessed as strong expression in the given scaffold group, and average scores of 2.3–3.4, 1–2.2, 0.1–0.9 were regarded as moderate, mild and minimal expression as previously outlined in detail (Knabe et al., 2008a; Knabe et al., 2017b; Knabe et al., 2017c). Also for assessing the density of the positively stained blood vessels an analogous semi-quantitative scoring system was employed, in which a score of (**+++**[ = 3]), (**++**[ = 2]), and (**+**[ = 1) denoted high, moderate or low blood vessel density, and a score of (0) absence of positively stained blood vessels. An average score of 2.5–3 was then assessed as high density in the respective experimental group, and an average score of 1.6–2.4 as moderate, an average score of 1–1.5 as slight, and an average score of 0.1–0.9 as minimal density.

### Statistical analysis

For statistical analysis the Kruskal–Wallis and Conover-Iman tests were utilized for multiple comparisons employing StatsDirect (version 3.0.150) software. Statistical significance was considered achieved for *p* < 0.05 and the data were expressed as mean ± standard deviation.

## Results

### Angio µ-CT analysis

**FIGURE 4:**
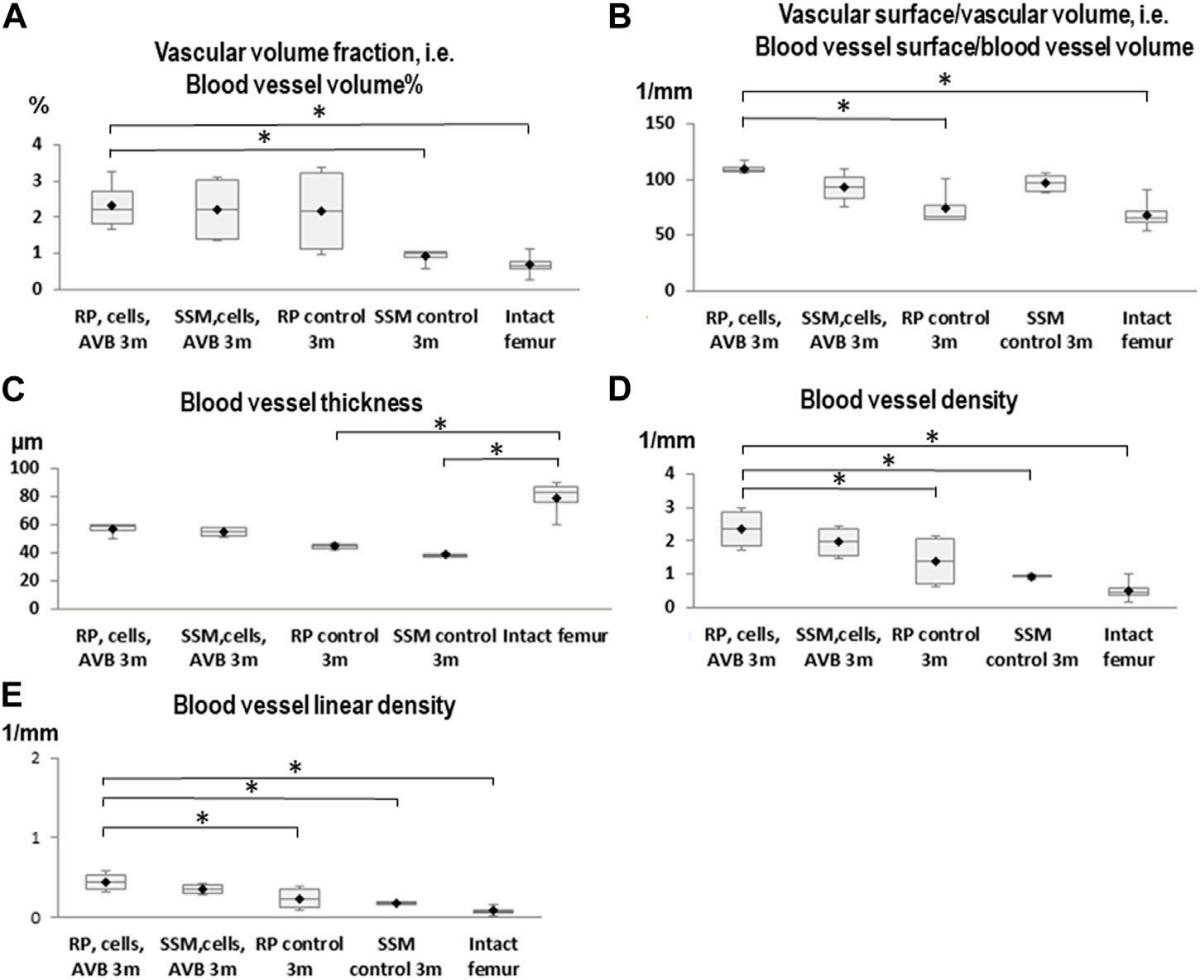
Box plot diagrams depicting the results of the angio-µCT analysis after 3 months of implantation. **(A)** Blood vessel volume%, **(B)** Blood vessel surface/blood vessel volume, **(C)** Blood vessel thickness, **(D)** Blood vessel density, **(E)** Blood vessel linear density in defects, in which RP, cells & AVB; SSM, cells & AVB, RP controls or SSM controls were implanted for 3 months. (*p < 0.05, **p < 0.001, ***p < 0.0001). Intact collateral femurs served as reference. The centerline represents the median. Half of the scores are less than the median and half are larger than the median. The two ends of the box show the range within which the middle 50% of all measurements lie, and the whiskers indicate the range within which the middle 75% of all measurements lie.

After 3 months of implantation a significantly greater blood vessel volume% was noted in defects reconstructed with RP, cells & AVB compared to those grafted with the SSM control (*p* ≤ 0.005), and compared to the intact femur (*p* ≤ 0.0004). Regarding blood vessel volume, blood vessel surface/volume and blood vessel thickness the differences were not statistically significant when comparing RP, cells & AVB to SSM, cells & AVB, SSM controls or to the intact femur, while blood vessel surface/volume was significantly greater in defects reconstructed with RP, cells & AVB compared to those grafted with RP controls (*p* ≤ 0.0001) or compared to the intact femur (*p* ≤ 0.0001). Defects reconstructed with either RP controls or SSM controls, i.e., without MSCs and AVB, displayed a significantly lower blood vessel thickness than the intact femur. Defects reconstructed with RP, cells & AVB to displayed a significantly higher blood vessel linear density than defects, in which RP controls (*p* ≤ 0.002) or SSM controls scaffolds (*p* ≤ 0.0078) were inserted, or than the intact femur (*p* ≤ 0.0001). Differences in blood vessel density were not statistically significantly different when comparing the RP, cells & AVB group to the SSM, cells & AVB group, while blood vessel density in the RP, cells & AVB group was significantly higher than in the RP control group (*p* ≤ 0.0302) or SSM control group (*p* ≤ 0.0154), or than in the intact femur (*p* ≤ 0.0001) (Figure 4; Table 1).

**TABLE 1:** Results of the angio-μCT analysis (mean + SD) at 3 months and at 6 months in defects grafted with the four different scaffold configurations.

Parameter	RP, cells & AVB 3m	RP, cells & AVB 6m	SSM, cells & AVB 3m	SSM, cells & AVB 6m	RP control 3m	RP control 6m	SSM control 3m	SSM control 6m	Intact femur
**blood vessel volume % [%]**	2.20 ± 0.49	2.79 ± 0.93	2.22 ± 1.24	1.25 ± 0.96	2.17 ± 1.42	1.12 ± 0.34	0.90 ± 0.19	0.78 ± 0.018	0.69 ± 0.43
**Blood vessel surface/volume [1/mm]**	$\underline{106.40\pm 4.45}$	$\underline{211.98\pm 12.80}$	92.60 ± 24.80	107.60 ± 6.44	$\underline{63.6\pm 0.34}$	$\underline{92.80\pm 0.85}$	102.90 ± 20.40	101.50 ± 8.30	69.70 ± 18.40
**Blood vessel thickness [µm]**	$\underline{57.00\pm 4.80}$	$\underline{88.00\pm 1.00}$	54.00 ± 2.10	57.00 ± 3.00	44.00 ± 2.00	54.00 ± 6.00	39.00 ± 1.60	52.00 ± 05.00	76.00 ± 9.00
**Blood vessel density [1/mm]**	2.35 ± 0.62	3.03 ± 0.67	$\underline{1.90\pm 0.60}$	$\underline{1.31\pm 0.95}$	1.38 ± 0.90	1.04 ± 0.30	0.92 ± 0.01	0.79 ± 0.04	0.53 ± 0.44
**Blood vessel linear density [1/mm]**	0.48 ± 0.16	0.73 ± 0.22	$\underline{0.35\pm 0.07}$	$\underline{0.27\pm 0.19}$	0.24 ± 0.15	0.19 ± 0.06	0.18 ± 0.02	0.14 ± 0.01	0.09 ± 0.06

(Statistical significant differences between 3 and 6 months for a given parameter appear in bold dark red print).

**FIGURE 5:**
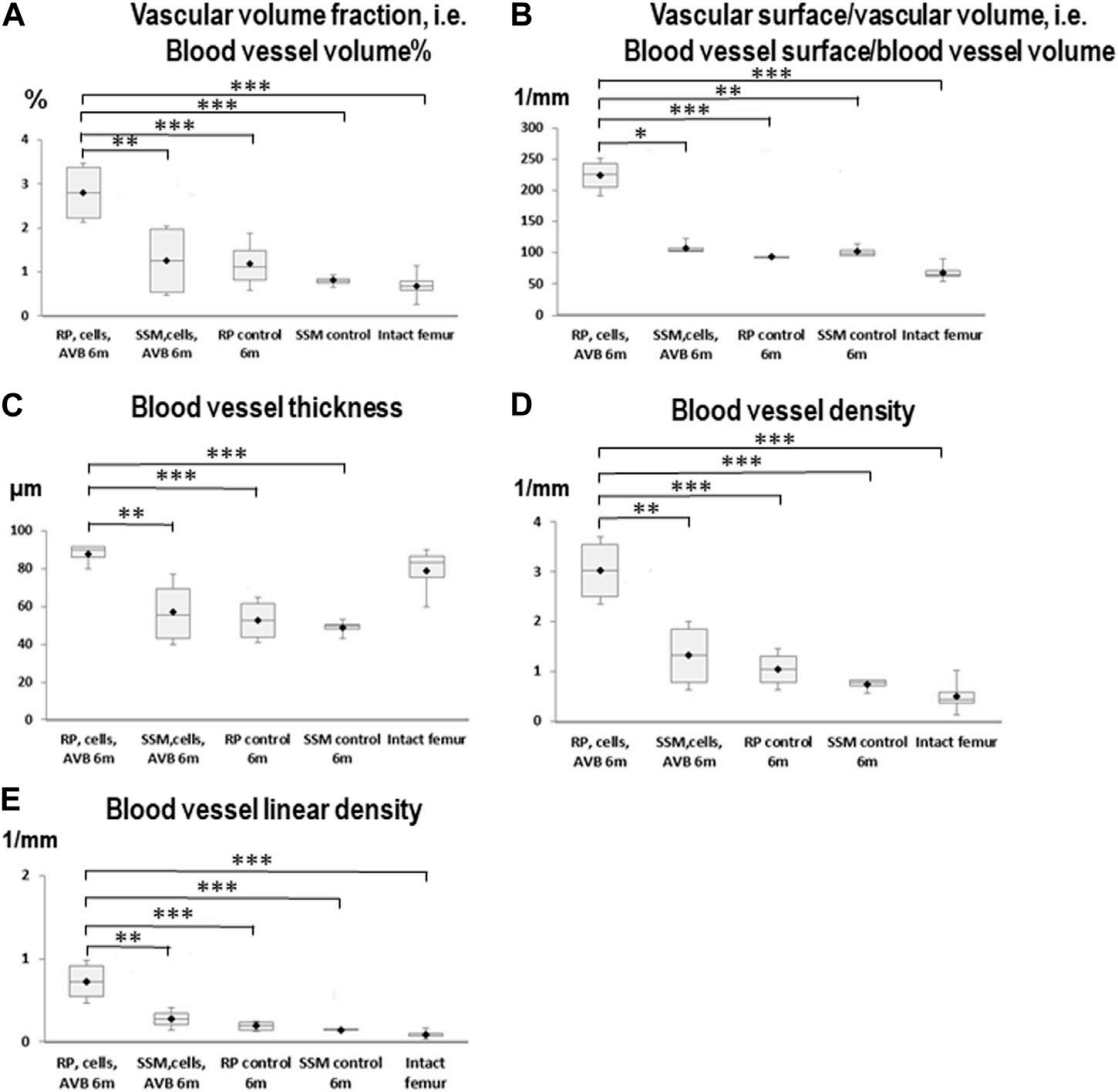
Box plot diagrams depicting the results of the angio-µCT analysis after 6 months of implantation. **(A)** Blood vessel volume%, **(B)** Blood vessel surface/blood vessel volume, **(C)** Blood vessel thickness, **(D)** Blood vessel density, **(E)** Blood vessel linear density in defects, in which RP, cells & AVB; SSM, cells & AVB, RP controls or SSM controls were implanted for 6 months. Intact collateral femurs served as reference (*p < 0.05, **p < 0.001, ***p < 0.0001). The centerline shows the median. Half of the scores are less than the median and half are larger than the median. The two ends of the box show the range within which the middle 50% of all measurements lie, and the whiskers represent the range within which the middle 75% of all measurements lie.

After 6 months of implantation defects reconstructed with RP, cells & AVB displayed a statistically significant higher blood vessel volume %, blood vessel surface/volume, blood vessel thickness, blood vessel linear density, and blood vessel density than defects grafted with SSM, cells & AVB, RP controls or SSM controls (*p* < 0,05). The same was true when comparing the RP, cells & AVB group to the intact femur, except for blood vessel thickness for which the difference was not statistically significant. (Figure 5; Table 1).

There was no significant increase in blood vessel volume% when comparing defects treated with RP, cells & AVB after 3 months to those after 6 months (Table 1). The same was true when comparing defects with RP controls or SSM controls at 3 months to those after 6 months. However, there was a significant increase in blood vessel volume noted when comparing defects grafted with SSM, cells & AVB at 3 months to those after 6 months of implantation (*p* ≤ 0.02). There was a significant increase of blood vessel surface/volume when comparing defects with RP, cells & AVB after 3 months to those after 6 months; the same was true when comparing defects with RP controls after 3 months to those after 6 months. Defects reconstructed with RP, cells & AVB also showed a significant increase of blood vessel thickness from 3 to 6 months (*p* ≤ 0.0001), while there was no significant increase in blood vessel density or in linear density from 3 to 6 months. Defects grafted with SSM, cells & AVB, on the other hand, showed a statistically significant decrease in blood vessel density (*p* ≤ 0.04) and linear density (*p* < 0.05) from 3 to 6 months (Table 1).

### Immunohistochemical analysis of angiogenic marker expression and vascular density level scoring

**FIGURE 6:**
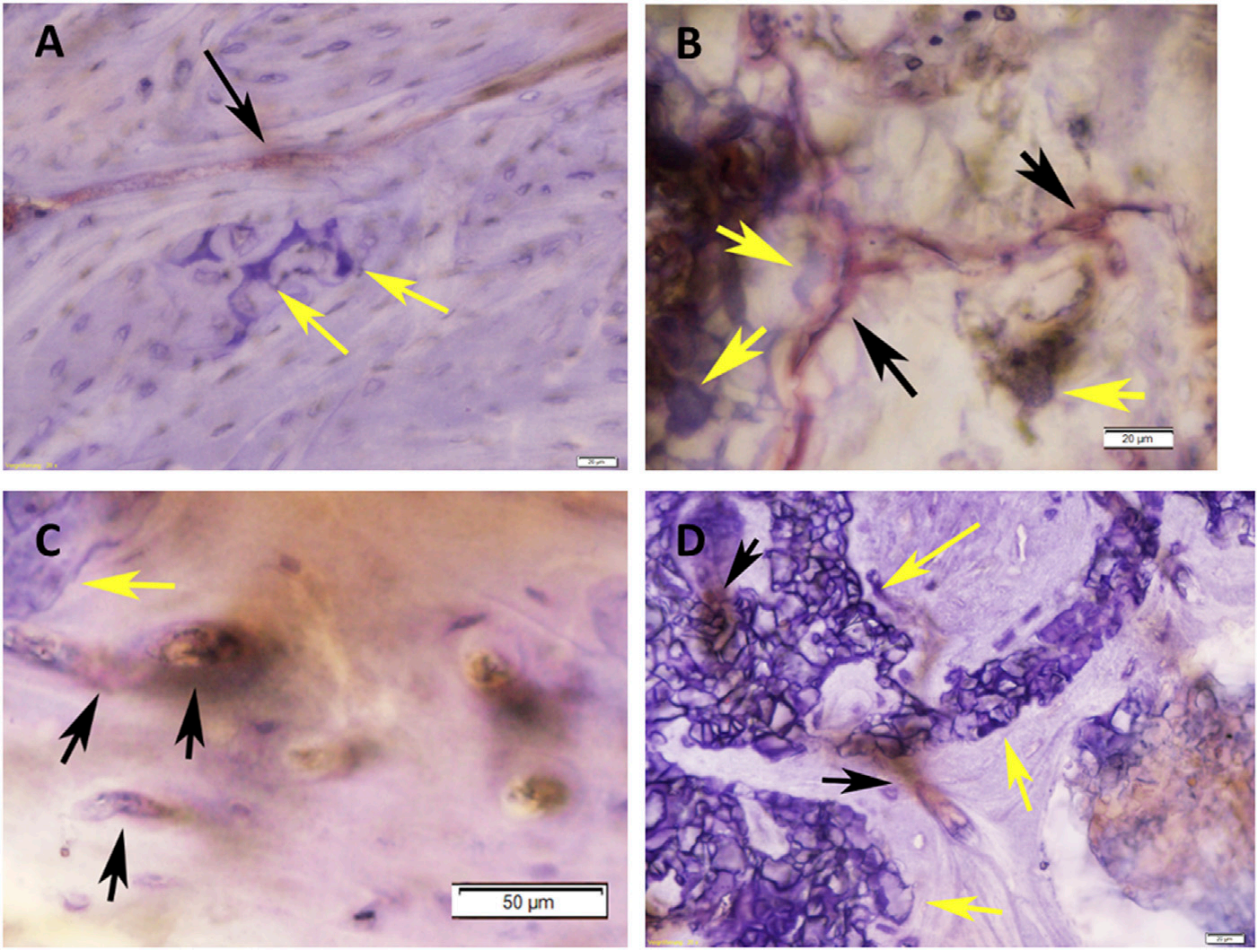
Histomicrographs of deacrylated sawed sections immunohistochemically stained for Von-Willebrand-Faktor (vWF). **(A)** Bone tissue which has formed after 3 months of implantation in a defect grafted with a RP, cells & AVB scaffold. A blood vessel with moderate to strong staining for vWF (black arrow) is visible in the vicinity of residues of the highly degraded silica containing calcium alkali orthophosphate (Si-CAOP) bioceramic scaffold material, which display excellent bone bonding behavior (yellow arrows), bar = 20 μm; **(B)** bone tissue formation after 3 months of implantation of a SSM, cells & AVB scaffold in the rat femoral segmental defect. Blood vessels with strong staining for vWF (black arrows) are present in the vicinity of the residues of the degraded Si-CAOP bioceramic scaffold material (yellow arrows), bar = 20 μm; **(C)** histomicrograph showing bone tissue which has formed after 3 months of implantation of a RP control scaffold in a segmental defect in the rat femur. Blood vessels with positive mild staining for vWF (black arrows) are visible adjacent to residues of the degraded Si-CAOP bioceramic scaffold material (yellow arrow), bar = 50 μm; **(D)** Histomicrograph showing blood vessel formation with mild to moderate staining for vWF (black arrows) in bone tissue which has invaded the pores of a degrading SSM control scaffold (yellow arrows—bioceramic scaffold material displaying good bone bonding) after 3 months of implantation, bar = 20 µm.

After 3 months of implantation, in defects grafted with RP, cells & AVB the highest blood vessel density scores of 2.5 for Col IV, 2.6 for vWF and 2.25 for CD 31 were noted among the various scaffold configurations, followed by defects, in which SSM, cells & AVB were used, with scores of 1.75 for vWF and CD31, and 1.5 for Col IV (Table 2). Defects with RP control and SSM control scaffolds displayed lower scores of 1 and 0.8, respectively, for CD 31; 0.5 and 0.4, respectively, for vWF; and scores of 1.5 and 1.33, respectively, for Col IV, corresponding to a minimal or slight density of positively stained blood vessels (Table 2; Figure 6).

**TABLE 2:** Results of the collagen IV, CD31 and vWF immunoscoring for the vascular density levels in defects grafted with the four different scaffold configurations.

Marker	Period	RP, cells & AVB	SSM, cells & AVB	RP control	SSM control
Collagen IV	l m	2.00	1.00	1.00	1.00
	3 m	2.50	1.50	1.50	1.33
	6 m	2.75	2.00	2.00	2.00
CD31	l m	2.00	1.00	1.00	0.60
	3 m	2.25	1.75	1.00	0.80
	6 m	2.75	2.00	2.00	1.00
vWF	l m	2.00	1.60	1.60	1.50
	3 m	2.60	1.75	050	0.40
	6 m	2.60	2.50	1.50	1.30

An average score of 2.50–3.60 was evaluated as high density of positively stained blood vessels in the given scaffold group, whereas an average score of 1.60–2.49, 1.00–1.59, and 0.10–0.90 was assessed as moderate, slight and minimal blood vessel density, respectively. vWF, von Willebrand factor.

At 6 months, defects with RP, cells & AVB displayed a score of 2.75 for collagen IV and CD 31, and 2.6 for vWF, which corresponded to a high density of positively stained blood vessel, while in defects with SSM, cells & AVB slightly lower scores of 2.5 for vWF and 2 for Col IV and CD 31 were noted (Table 2). Defects with RP controls yielded a lower score of 1.5 for vWF and similar scores for Col IV and CD 31, followed by defects with SSM controls with a score of 2 for Col IV, and 1.3 and 1 for vWF and CD31, respectively (Table 2).

### Results of the histomorphometric analysis

#### Bone area fraction

**FIGURE 7:**
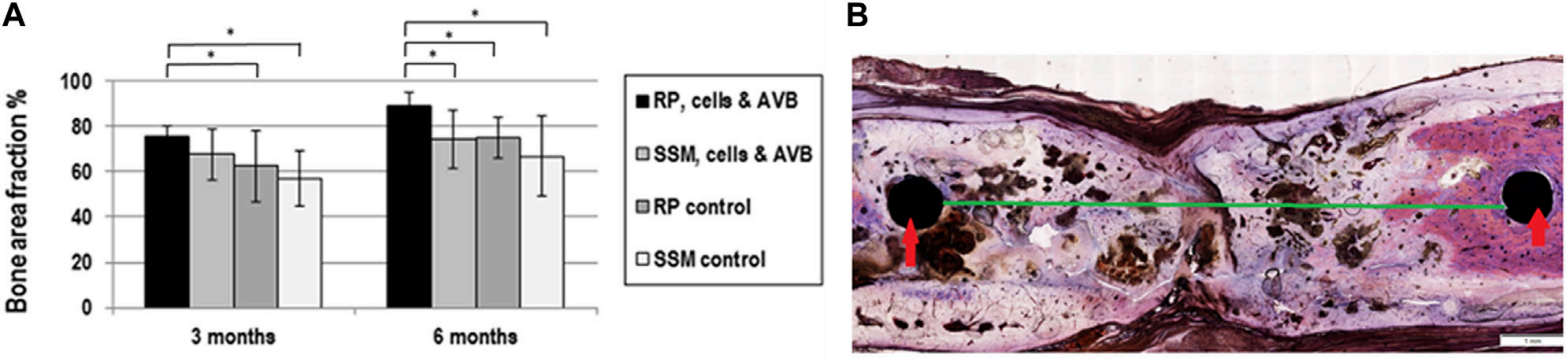
**(A)** Histogram outlining the result of the histomorphometric measurements of the bone area fraction, in defects grafted with RP, cells & AVB; SSM, cells & AVB, RP controls or SSM controls after 3 and 6 months of implantation (*p < 0.05). All values are mean ± standard deviation of 5 measurements. **(B)** Histomicrograph of deacrylated sawed sections of segmental defect after 6 months of implantation of a RP, cells & AVB scaffold showing bridging of the rat femoral defect, fixation screws (red arrows); 6 mm defect (green line).

At 3 months, the greatest bone area fraction, i.e., greatest bone formation, was noted in defects grafted with RP, cells & AVB (75.8%, mean) followed by defects grafted with SSM, cells & AVB (67%, mean), then defects with RP controls (62%), and finally in defects with SSM controls (57%, mean). These differences in bone area fraction were statistically significant, when comparing defects reconstructed with RP, cells & AVB at 3 months to defects grafted with RP controls (*p* ≤ 0.01) or SSM controls (*p* ≤ 0.0008). The differences in bone area fraction at 3 months, when comparing defects with RP, cells & AVB to those with SSM, cells & AVB, were, however, not statistically significant, and only showed a trend toward greater bone formation (*p* ≤ 0.07) (Figure 7A).

After 6 months, defects grafted with 3D printed scaffolds, cells & AVB displayed defect bridging (Figure 7B) and the greatest amount of bone area fraction (89%, mean) followed by sites, in which 3D printed control scaffolds were used (75%, mean), then by defects with SSM scaffolds, cells & AVB (74%, mean) and finally by defects in which SSM control scaffolds were implanted (66%, mean) (Figure 5). These differences in bone area fraction were statistically significant, when comparing defects with RP, cells & AVB after 6 months of implantation to sites with SSM, cells & AVB (*p* = 0.03) or to defects with RP controls or SSM controls after 6 months (*p* = 0.03 and 0.01 respectively) (Figure 7A).

#### Scaffold bioceramic area fraction

**FIGURE 8:**
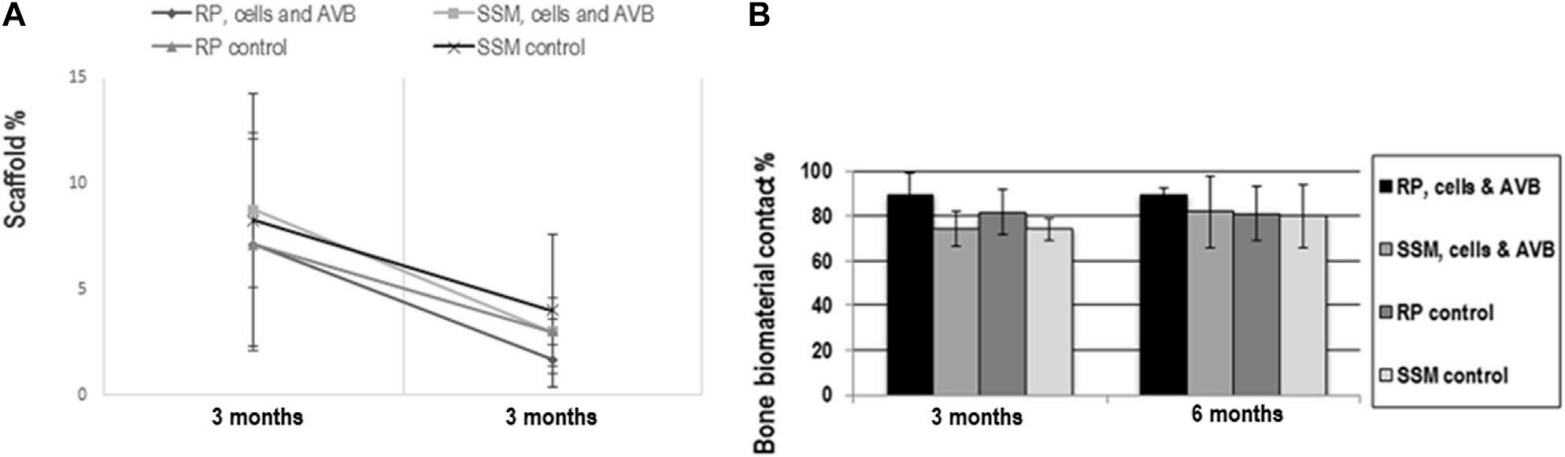
Results of histomorphometric measurements of scaffold area fraction **(A)** and bone biomaterial contact **(B)**: **(A)** line graph showing the change in scaffold area fraction in the 4 different experimental groups from 3 to 6 months. All values are mean ± standard deviation of 5 measurements. **(B)** Histogram showing the bone biomaterial contact percentage in defects grafted with RP, cells & AVB; SSM, cells & AVB, RP controls or SSM controls after 3 and 6 months of implantation. All values are mean ± standard deviation of 5 measurements.

A decrease in scaffold area fraction was noted between 3 and 6 months of implantation with all scaffold configurations, there, however, were no statistically significant differences in scaffold area fraction when comparing the four different scaffolds configurations to each other at 3 and 6 months of implantation (Figure 8A). Si-CAOP scaffolds of all test groups exhibited a high biodegradability after 6 months, which was highest with RP, cells & AVB, without this difference being statistically significant. This was, however, associated with greatest bone formation (Figure 7A).

#### Bone biomaterial contact

**FIGURE 9:**
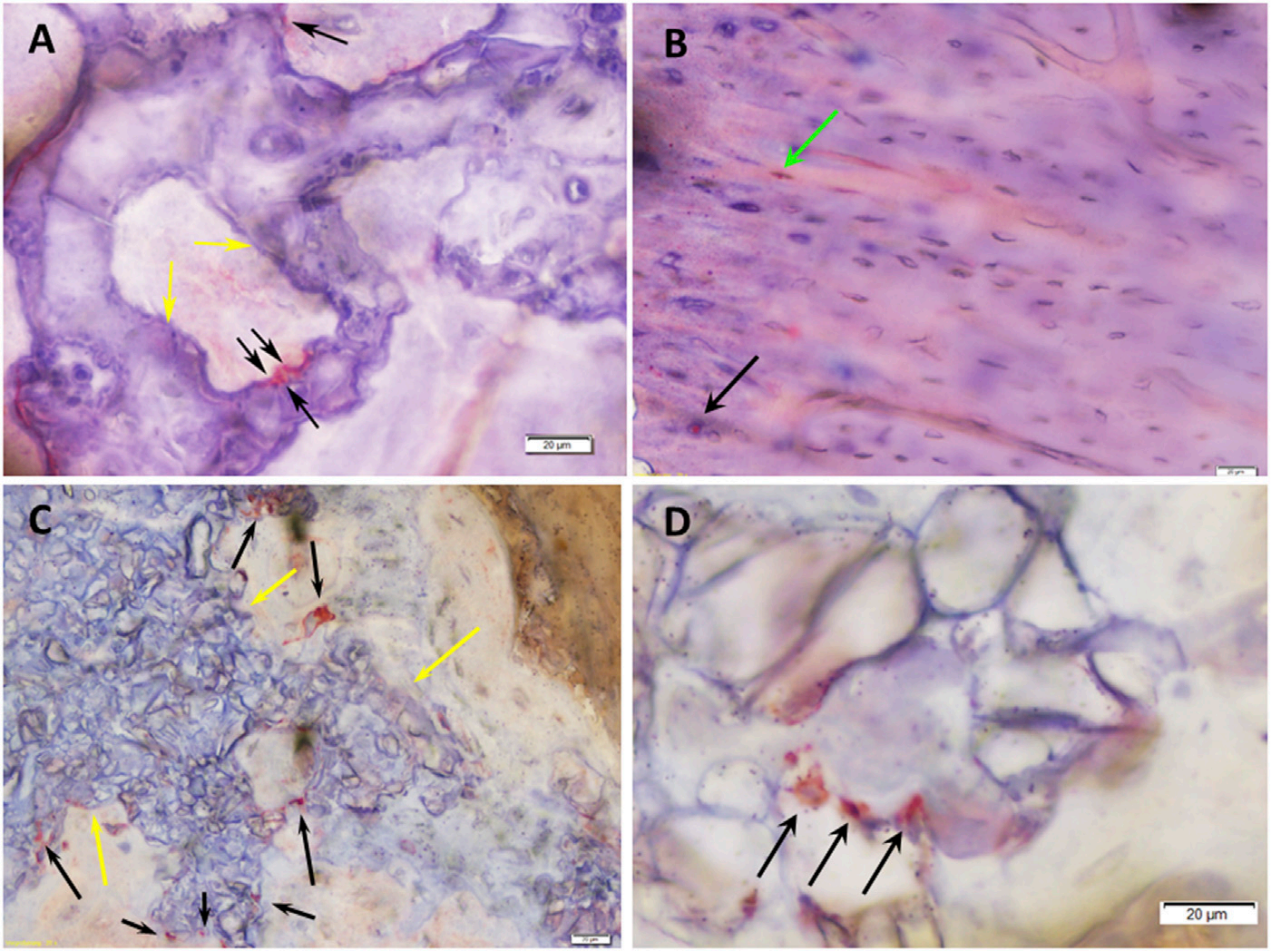
Histomicrographs of deacrylated sawed sections immunohistochemically stained for osteocalcin (OC). **(A)** Bone tissue which has formed after 3 months of implantation in a defect grafted with a RP, cells & AVB. The highly leached Si-CAOP bioceramic exhibits excellent bone biomaterial contact, i.e., bone bonding behavior (yellow arrows). Osteoblasts with strong osteocalcin expression (black arrows) are present at the surface of the degrading Si-CAOP material. This is indicative of ongoing matrix mineralization, bone formation and remodeling with the bioceramic material being gradually replaced by newly formed bone. **(B)** Lamellar bone which has formed after 6 months of implantation in a defect grafted with a RP, cells & AVB. Osteocytes (green arrow) and osteoblasts (black arrow) with strong osteocalcin expression are visible indicating areas with active bone remodeling. **(C)** Degrading and partially highly leached Si-CAOP ceramic material displaying excellent bone bonding (yellow arrows) after 3 months of implantation of an SSM control scaffold. Osteoblasts with strong osteocalcin expression (black arrows) are present at the surface and in the pores of the degrading Si-CAOP material indicating areas of progressing matrix mineralization and bone formation with the bioceramic material being gradually replaced by newly formed bone. **(D)** Highly leached residual Si-CAOP ceramic material after 6 months of implantation of an SSM control scaffold. The presence of osteoblasts with strong osteocalcin expression (black arrows) demonstrates actively progressing bone formation, which gradually replaces the Si-CAOP ceramic material, bar = 20 µm.

RP, cells & AVB displayed the highest bone biomaterials contact (bone bonding behavior) at 3 and 6 months, however, without this difference being statistically significant (Figure 9). Taken together, there was no significant difference in the bone biomaterial contact percentage when comparing the four different experimental groups to each other after 3 months and 6 months of implantation (Figure 8B). All scaffold configurations exhibited high bone biomaterial contact, i.e., excellent bone bonding behavior (Figures 6, 8B, 9) of the residual Si-CAOP material, with a tendency towards higher bone bonding for RP, cells & AVB.

### Immunohistochemical analysis of osteogenic marker expression

The results of the semi-quantitative scoring of the of the osteogenic marker expression of osteocalcin, bone sialoprotein, alkaline phosphatase and type I collagen in the various cell and matrix components of the bone tissue, which has formed in the segmental femoral defects 3 and 6 months after implantation of various scaffold configurations are given in Tables 3, 4. Defects, in which RP, cells & AVB were implanted, showed higher expression scores for most of the osteogenic markers in both osteoblasts and the mineralized bone matrix after 3 and 6 months of implantation when compared to the other scaffold configurations. In this they were followed by defects with SSM, cells & AVB (Tables 3, 4). Figure 9 demonstrates the excellent bioactive and bone bonding behavior of the residual Si-CAOP bioceramic material of the RP and SSM scaffolds. Osteoblasts with strong osteocalcin expression are visible at the surface of the degrading highly leached bioceramic material. This is indicative of ongoing matrix mineralization, bone formation and remodeling with the bioceramic material being gradually replaced by newly formed bone tissue.

**TABLE 3:** Immunoscoring results of the expression of osteocalcin, bone sialoprotein, alkaline phosphate and type I collagen in the various cell and matrix components of the bone tissue formed after 3 months of implantation of the various scaffold configurations.

Marker	Scaffold group	Osteoblasts	Osteocytes	Fibroblastic cells of the osteogenic mesenchyme	Fibous matrix	Bone matrix	Mineralizing matrix
OC	RP, cells & AVB	3.50	1.00	2.10	2.10	2.80	2.80
	SSM, cells & AVB	1.70	**0.00**	1.00	1.00	1.50	1.75
	RP control	1.60	**0.00**	2.00	1.00	2.00	2.00
	SSM control	2.60	**0.00**	1.60	1.50	2.00	2.25
BSP	RP, cells & AVB	3.20	1.00	2.40	2.40	3.20	3.20
	SSM, cells & AVB	1.70	**0.00**	1.00	1.00	1.50	3.00
	RP control	1.60	**0.00**	1.60	1.00	2.00	2.00
	SSM control	2.70	**0.00**	2.00	2.00	2.00	1.75
ALP	RP, cells & AVB	3.25	1.00	2.00	2.50	2.25	3.50
	SSM, cells & AVB	2.75	0.25	3.00	3.00	1.75	2.50
	RP control	1.60	**0.00**	1.50	1.50	1.00	2.30
	SSM control	0.50	**0.00**	1.50	1.50	0.50	0.50
Col 1	RP, cells & AVB	3.30	1.00	2.30	2.30	3.00	3.60
	SSM, cells & AVB	3.30	0.60	2.30	2.30	2.30	3.60
	RP control	1.60	0.60	1.50	1.60	1.00	1.75
	SSM control	1.25	**0.00**	1.60	1.60	1.00	1.60

An average score of 3.50–5.00 was evaluated as strong staining in the given scaffold group, whereas an average score of 2.30–3.49, 1.00–2.29, and 0.10–0.99 was assessed as moderate, mild and minimal staining, respectively. Col I, type I collagen; ALP, alkaline phosphatase; OC, osteocalcin; BSP, bone sialoprotein.

**TABLE 4:** Immunoscoring results of the expression of osteocalcin, bone sialoprotein, alkaline phosphate and type I collagen in the various cell and matrix components of the bone tissue formed after 6 months of implantation of the various scaffold configurations.

Marker	Scaffold group	Osteoblasts	Osteocytes	Fibroblastic cells of the osteogenic mesenchyme	Fibous matrix	Bone matrix	Mineralizing matrix
OC	RP, cells & AVB	4.50	0.70	3.30	3.00	4.50	3.20
	SSM, cells & AVB	3.50	1.50	3.00	3.00	3.50	3.50
	RP control	3.60	1.50	1.00	1.00	3.60	1.30
	SSM control	3.50	1.00	2.00	2.00	2.50	3.00
BSP	RP, cells & AVB	4.00	0.90	3.00	3.00	3.60	3.60
	SSM, cells & AVB	3.50	1.30	3.00	3.00	3.60	3.50
	RP control	3.60	1.00	1.00	1.00	3.50	1.30
	SSM control	3.50	**0.00**	3.00	3.00	2.00	3.00
ALP	RP, cells & AVB	2.00	0.25	3.00	1.00	3.00	3.00
	SSM, cells & AVB	2.00	1.00	2.00	2.00	3.00	2.00
	RP control	2.50	1.00	1.50	3.00	2.00	3.00
	SSM control	2.00	1.00	3.00	1.00	.002	3.00
Col 1	RP, cells & AVB	4.00	3.00	3.00	3.00	4.00	3.00
	SSM, cells & AVB	3.00	1.50	3.00	2.00	3.00	3.00
	RP control	2.00	1.00	1.00	1.00	2.00	3.00
	SSM control	1.00	1.00	0.50	0.50	1.50	1.50

An average score of 3.50–5.00 was evaluated as strong staining in the given scaffold group, whereas an average score of 2.30–3.49, 1.00–2.29, and 0.10–0.99 was assessed as moderate, mild and minimal staining, respectively. Col I, type I collagen; ALP, alkaline phosphatase; OC, osteocalcin; BSP, bone sialoprotein.

## Discussion

Even when using 3 D bioceramic scaffolds, whose material properties and architecture enhance bone formation, for bone tissue engineering, failure to ensure sufficient blood supply for the tissue engineered construct will most likely lead to a compromised outcome. As such, vascularization is crucial for bone repair of large discontinuity defects. In this context, creating an AVL or AVB using microvascular surgery is a reliable approach for achieving intrinsic vascularization, which is underscored by the findings reported by Arkudas et al. (2015), who demonstrated that between 10 and 14 days after implantation of an AVL direct luminal sprouting occurred from the femoral vein and that more than 90% of their scaffold were vascularized 8 weeks after implantation of the AVL. In addition, improved osteoblasts survival due to intrinsic vascularization was shown (Arkudas et al., 2007; Polykandriotis et al., 2009). To our knowledge, the methodology outlined in this paper is a novel approach for characterizing the vascularization of a bone tissue engineered construct and scaffolds after 3 and 6 months of *in vivo* implantation entailing the use of an AVB in a comprehensive manner. The overall objective of our study was to explore an advanced bone tissue engineering approach, combining 3D powder bed printed bioactive resorbable bioceramic scaffolds with an advanced perfusion flow cell culture technique for pre-colonization of these scaffolds with mesenchymal stem cells, and with an advanced intrinsic AVB angiogenesis technique, for repair of critical size, segmental discontinuity defects in a femoral rat model. To this end, the specific aims of this study entailed evaluating the effect of 3D printed bioactive Si-CAOP scaffolds endowed with homogenously distributed osteoblasts and mineralizing bone matrix by pre-colonization and implanted with an AVB, on the neovascularization of the tissue engineered construct and on bone formation and repair of these critical-size segmental defects after 3 and 6 months as compared to utilizing scaffolds manufactured by the conventional SSM replica technique. To avoid poor vascularization in the center of the tissue engineered construct resulting from slow blood vessel ingrowth from the native bone adjacent to the defect, as is common with the extrinsic vascularization mode, microvascular surgery was applied for creating an AVB which was placed in the center of the 3D printed or the SSM scaffolds pre-colonized with mesenchymal stem cells during *in vivo* implantation to achieve intrinsic vascularization of the tissue engineered constructs with capillary formation spreading from the center of the discontinuity defect towards the periphery (Yang et al., 2013).This approach was in line with a study that also utilized an AVB technique and reported that tissue engineered bone pre-vascularized with a saphenous arteriovenous vascular bundle was capable of enhancing osteogenesis leading to successful repair of fibular defects in beagle dogs (Cai et al., 2011). In our study, for quantification of blood vessels formed in the defects grafted with the different scaffold types with and without cell pre-colonization and AVB, first, a sequence of intravascular contrast enhancement steps was applied followed by reduction of X-ray absorption of the bone samples. Subsequently, 3D morphometry was performed to characterize the distribution pattern of the microvasculature (Kampschulte et al., 2016). After 6 months of implantation defects reconstructed with RP, cells & AVB exhibited a significantly higher blood vessel volume percentage (mean 2.79%) compared to defects grafted with SSM, cells & AVB (mean 1.25%). This may be related to the fact that 3D printed scaffolds displayed a significantly greater amount of homogenously distributed macropores larger 600 µm in size (32.8% of pores) than the SSM scaffolds with only 8.8% of their pores having a size of more than 600 µm. This would be in agreement with findings reported by Bai et al. (2010) who reported that macropores larger than 400 μm supported angiogenesis and osteogenesis progression, and were suitable for bone tissue engineering applications (Bai et al., 2010). With the RP, cells & AVB group, there was an increase of blood vessel volume% from 2.20% to 2.79% from 3 to 6 months, while with the SSM, cells & AVB group a decrease from 2.22% to 1.25% occurred from 3 to 6 months. This may be due to the specific distribution of pore sizes in 3D printed scaffolds, which may favor the branching of blood vessels, which gradually increased from three to 6 months, while the pore configuration of SSM scaffolds may have limited blood vessel development early on. Moreover, at 6 months, defects in which RP, cells & AVB were implanted, displayed the highest blood vessel surface per volume (211.98 mean), which is a measure for the complexity of the vasculature, whereas a mean value of 107.6 was noted in defects grafted with SSM, cells & AVB. This was associated with a higher increase in blood vessel surface per volume from 106.4 (mean) at 3 months to 211.98 (mean) at 6 months in defects reconstructed with RP, cells & AVB compared to defects, in which SSM, cells & AVB were placed, for which an increase from 92.6 (mean) at 3 months to 107.6 (mean) at 6 months was noted. This may be related to a greater degree of capillary branching occurring from 3 to 6 months in defects, in which RP, cells & AVB were utilized. Defects with RP, cells & AVB also exhibited a greater blood vessel thickness (88 µm mean) than defects with SSM, cells & AVB (57 µm mean) after 6 months with blood vessel thickness increasing from 57 μm to 88 µm between 3 and 6 months for RP, cells & AVB, while in defects grafted with SSM, cells & AVB blood vessel thickness did not increase significantly between 3 and 6 months of implantation. The finding of increase in blood vessel thickness in defects reconstructed with RP, cells & AVB is in agreement with findings reported by Polykandriotis et al. (2009), who when employing an intrinsic vascularization approach observed an increase in diameter of blood vessels after 2 months so as to accommodate increased perfusion from arterioles, precapillary arterioles, capillaries and postcapillary venules, to draining venules and veins. Defects with RP, cells & AVB furthermore displayed a higher blood vessel density and a higher linear blood vessel density after 6 months of implantation than SSM, cells & AVB, than RP controls, and SSM controls. Blood vessel density and linear blood vessel density in defects with RP, cells & AVB increased between 3 and 6 months, however, without this difference being statistically significant. In contrast, in defects, in which SSM, cells & AVB were utilized, blood vessel density and linear blood vessel density decreased between 3 and 6 months. This may be related to greater blood vessel branching occurring in defects, in which RP, cells & AVB were implanted, which then led not only to a higher blood vessel density but also to a linear pattern resulting in a more uniform vascularization throughout the entire defect site. In contrast, defects grafted with SSM, cells & AVB exhibited a lower degree of vascularization, and the blood vessels within the defects grafted with SSM, cells & AVB did not branch from 3 to 6 months, which was associated with significantly lower bone formation.

Angio-µCT was used for these quantitative measurements. In addition, immunohistochemical analysis was used to visualize the blood vessel density within the scaffolds and to study their interaction with bone cells and matrix as well as their formation in conjunction with the formation of the bone tissue. The first marker used for the immunohistochemical analysis was collagen IV, since the major two components of blood vessels basal lamina are collagen-type IV and laminin. Collagen IV is deposited by endothelial cells in layers in the basement membrane (Schlingemann et al., 1991). Defects grafted with RP, cells & AVB showed the highest collagen IV expression, especially after 3 and 6 months with scores of 2.5 and 2.75, respectively. The other 3 groups displayed lower collagen IV expression. This indicates that reconstruction of the segmental defects with RP, cells & AVB resulted in greatest blood vessel proliferation and the most mature vasculature. This finding is in agreement with the fact that pericytes induce the production of the collagen IV matrix, which initiates migration of endothelial cells, formation of cell-cell contacts, maturation and vascular stabilization (Zhou et al., 2016). CD31 is a transmembrane glycoprotein located at the cell-cell junction of endothelial cells which are homotypic contacts and associated with cell adhesion. Homotypic contacts are important for blood vessels formation and maintenance. vWF is synthesized by endothelial cells and megakaryocytes and is considered an important marker protein for the immunohistochemical study of angiogenesis (Newton et al., 1999; Simon and McWhorter, 2002; Pusztaszeri et al., 2006). Defects with RP, cells & AVB displayed the highest score of blood vessel density with positive CD31 expression after 3 and 6 months 2.25 and 2.75, respectively. The other 3 defect groups displayed lesser values. It was shown in a previous study (Adel-Khattab et al., 2018) that 3D printed RP scaffolds displayed a higher percentage of macropores ≥600 µm than SSM scaffolds. Thus our findings for the RP, cells & AVB group were in good correspondence with findings of a study on calcium phosphate cements (CPC) with a macropore range 50–800, average pore size is 490 μm, which reported the self-assembly of endothelial cells in macropores resulting in organization of microcapillary-like structures with multiple sprouts and tube-like structures, whose lumens were branched and interconnected. It furthermore was shown that the prevascular network in these macroporous CPCs was highly interconnected. PECAM1, collagen I, and vWF immunohistochemical assays and SEM examination revealed highly extensive interconnected vascular-like structures on CPC (Thein-Han and Xu, 2013). Both, at 3 and 6 months, defects grafted with RP, cells & AVB displayed the highest density score for vessels with positive vWF expression, which is indicative of a highly proliferative vasculature resulting from the use of the AVB technique in combination with 3D printed RP scaffolds. This is in correspondence with evidence that early endothelial progenitor cells express surface markers such as CD14, CD45, and CD133, while, late endothelial progenitor cells express CD31 and vWF, and which points towards a high proliferative potential with respect to incorporation into the resident vasculature (Shantsila et al., 2008; Costa-Almeida et al., 2014). It furthermore has been shown that silicon doping of 3D printed TCP scaffolds had an enhancing effect both on osteogenesis and angiogenesis (Bose et al., 2017). Previously, we were able to demonstrate that 3D printed RP scaffolds exhibited a greater selective silicon ion release than SSM scaffolds (Adel-Khattab et al., 2018). Consequently, in addition to the difference in microarchitecture and pore size distribution, the greater silicon ion release may also have contributed to the more mature vasculature and greater bone formation observed in our current study when using RP, cells & AVB compared to using SSM, cells & AVB for repair of segmental discontinuity defects. This aspect merits further investigation employing adequate endothelial cell and osteoblast-endothelial cell co-culture studies (Unger et al., 2007; Fuchs et al., 2009).

The histomorphometric analysis of the critical size defects revealed that a greater amount of bone area fraction was present in defects grafted with RP, cells & AVB, i.e. 75% and 89% at 3 and 6 months of implantation followed by defects, in which RP controls without cells were used, i.e. 62% and 75% of bone area fraction at 3 and 6 months, then defects with SSM, cells & AVB, which displayed a bone area fraction of 67% and 70% after 3 and 6 months of implantation, respectively, and finally the sites grafted with the SSM controls, which exhibited 55% and 66% of bone area fraction at 3 and 6 months, respectively. Defects grafted with RP, cells & AVB also displayed the highest osteogenic marker expression. This may be related to the fact that in the previous *in vitro* study (Adel-Khattab et al., 2018) 3D printed scaffolds displayed the greatest formation of osteoblasts and mineralizing bone matrix elaborated by these cells 1 week after perfusion culture of mesenchymal stem cells, due to exhibiting a highly organized microporosity and interconnected macropores, which favor bone organization and mimic properties of the native bony extracellular matrix. The bone formation pattern in both 3D printed RP scaffolds and SSM scaffolds showed initiation of bone formation on the surface of the material as well as in the pores by formation of bony islands followed by further continuously progressing scaffold leaching and degradation in combination with deposition of osseous tissue inside the increasing scaffold pores and on the surfaces of the scaffolds. The high bone-biomaterial contact, i.e., excellent bone bonding behavior, and high biodegradability noted with RP, cells & AVB was in accordance with findings for Si-CAOP granules in the sheep sinus floor and in the human case (Knabe et al., 2017a,e). The fact that the differences in bone-biomaterial contact, and scaffold area fraction between the different groups were not statistically different, may be related to the high biodegradability of the Si-CAOP scaffolds and the limited residual scaffold material after 3 and 6 months. Greatest bone formation and highest osteogenic marker expression was noted in defects grafted with RP, cells & AVB, with bridging between the two ends of the critical size defects (Rack et al., 2020). And bone remodeling was still progressing at 6 months. This was associated with displaying a more mature vasculature. In this context, it needs to be emphasized that this outcome was achieved purely on the basis of excellent bioactive properties of the 3D printed Si-CAOP bioceramic scaffolds without having to add growth factors or other biologics, which would make regulatory approval costly and complicate translation to the clinic. In addition, when using growth factors such as VEGF or PDGF for regenerating bony defects in cancer patients the risk of inducing recurrence of the tumor or metastases cannot be fully ruled out (Sand et al., 2014). Furthermore, Ayoub et al. (2016) showed that while a BMP-7 containing putty was successfully used for reconstruction of alveolar clefts in young patients, segmental defects in the mandible of elderly patients first appeared to heal but failed long-term. As a result, our advanced tissue engineering approach entailing combination of binder jet 3D powder bed printed Si-CAOP scaffolds with pre-colonization with mesenchymal stem cells and with the AVB technique, which in the current study was first tested in a well-established femoral segmental rat defect model, appears to be a promising approach for reconstructing segmental mandibular defects after tumor ablation. To advance this approach to the clinic, currently a preclinical large animal study, which utilizes a clinically relevant mini-pig model, is underway for patient-specific reconstruction of a critical size segmental mandibular defect employing a 3D printed Si-CAOP scaffold, mesenchymal stem cells and the AVL technique. This entails optimizing scaffold design for upscaling of the scaffold to anatomical relevant dimensions (Supplementary Figure S1), which also requires optimizing perfusion cell seeding and culturing conditions so as to achieve an ideal shear stress on the seeded mesenchymal stem cells during pre-colonization (Voronov et al., 2010; Pham et al., 2012). In addition, longer time periods of 9 and 12 months will be studied so as to be able to further follow the remodeling process of the bone microarchitecture.

In the present study, binder jet 3D powder bed printing was utilized for fabricating 3D Si-CAOP scaffolds. This yielded scaffolds and struts with high microporosity which resulted in a positive biological effect with respect to cell colonization and mineralizing extracellular matrix elaboration during *in vitro* pre-colonization and with respect to capillary and bone formation *in vivo*. Alternative 3D printing techniques such as stereolithography 3D printing result in scaffold struts with lower porosity which may not exhibit this positive biological effect. In this context, there have been efforts to develop adequate technologies for increasing the porosity of struts of ceramic scaffolds fabricated by stereolithography 3D printing (Ahlhelm et al., 2021).

In conclusion, angio-µCT and immunohistochemical and histomorphometric analysis demonstrated that the intrinisic angiogenesis AVB technique is a well suited methodology for achieving adequate vascularization of the 3D printed scaffolds and respective tissue engineered constructs after 3 and 6 months of implantation in critical size segmental discontinuity defects and that our tissue engineering approach employing binder jet 3D powder bed printed RP scaffolds facilitated segmental defect repair. As such, defects grafted with RP, cells & AVB exhibited a higher degree of mature vasculature and bone regeneration than defects in which SSM, cells & AVB were used.

As a result, 3D powder bed printed Si-CAOP ceramic scaffolds hold great promise for repair of critical size segmental discontinuity bone defects utilizing a bone tissue engineering approach, which entails scaffold colonization with mesenchymal stem cells and use of the AVB vascularization technique. This is in addition to offering the possibility of producing a synthetic bioceramic patient specific bone graft on the basis of the patient’s CT data. Consequently, proof of concept has been achieved, and a preclinical large animal study employing a clinically representative defect model is underway for evaluating this approach for patient specific reconstruction of large mandibular segmental bone defects, which represents the next step towards translation to the clinical arena in the context of this overall translational research program.

## Data Availability

The original contributions presented in the study are included in the article/Supplementary Material, further inquiries can be directed to the corresponding author.
