# Supplementary material for: A tissue engineered 3D printed calcium alkali phosphate bioceramic bone graft enables vascularization and regeneration of critical-size discontinuity bony defects in vivo
Source: Front Bioeng Biotechnol. 2023 Jun 15;11:1221314. doi: 10.3389/fbioe.2023.1221314 (PMC10311449; doi:10.3389/fbioe.2023.1221314)
Supplement: Supplementary file 1 [file DataSheet1.PDF]

Supplementary material

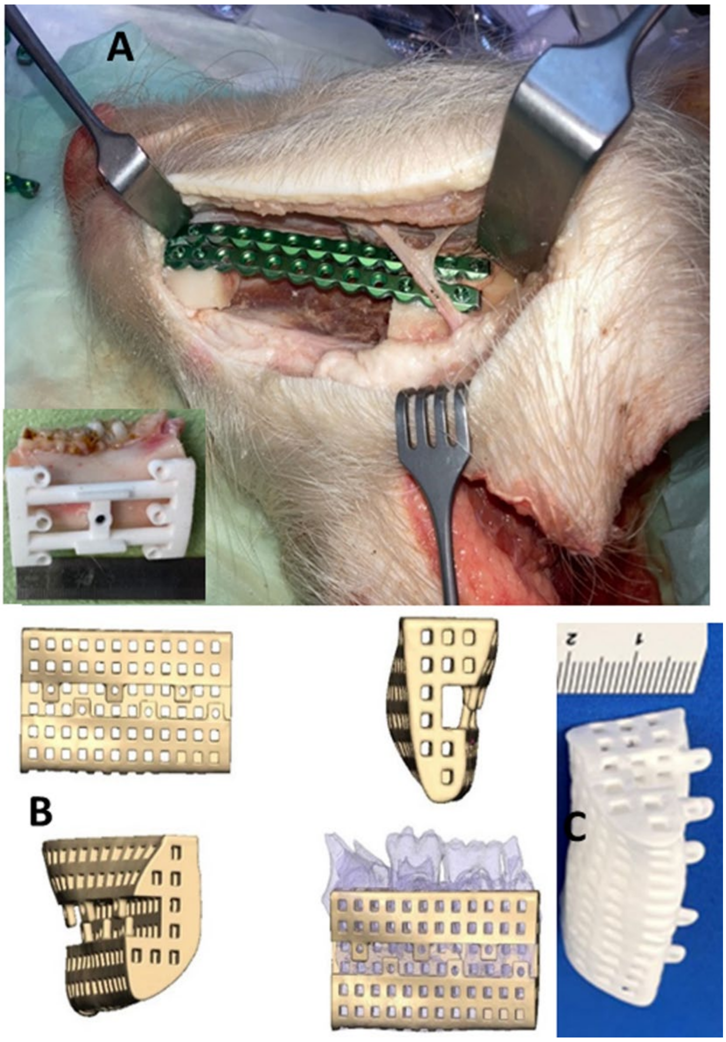

FIGURE S1. Design of preclinical mini-pig model for evaluating patient specific binder jet 3D powder bed printed scaffolds in combination with mesenchymal stem cell pre-colonization and an intrinsic angiogenesis approach for reconstruction of large mandibular segmental bone defects. (A) critical-size segmental discontinuity defect in the mini-pig mandible, (B) schematic - scaffold design, (C) 3D powder bed printed Si-CAOP scaffold.

Rationale for first using a rat femoral model followed by a mandibular mini-pig model in the translation process.

First, a rat model was chosen in the context of a translational research program, so as to be able to examine the aspect of scaffold microarchitecture at varying time points in a sufficiently large number of animals and to provide a proof of concept for the chosen tissue engineering approach prior to advancing with the most suitable scaffold configuration to an extremely demanding large animal mini-pig model featuring a critical size segmental mandibular defect 5 cm in size. For this preclinical study, a smaller number of animals can then be used based on the preselection, which is in line with well-established animal ethics principles. Moreover, the well-established femoral rat model was used, since due to the small dimensions of the rat mandible creating a segmental defect in the horizontal body of the rat mandible is not easily possible. As a result, there is no biomechanically stable established

segmental model with respective osteosynthesis plates available for this anatomical location. In addition, the rat mandible is only 1-2 mm thick in this area, which does not allow for using a 3D printed scaffold with the combined macro- and microarchitectural features of the scaffolds examined in our study, for which a diameter of 5 mm was needed, in order to print scaffolds with these features. Both femoral and mandibular critical size segmental defect models are very demanding models for animal experimentation, among others with respect to achieving mechanically competent osteosynthesis. Both the femur and the mandible possess a relatively thick cortical bone layer in larger animals and in humans. In the mandible there is a layer of cancellous bone of less than 10 mm thickness located in between the lingual and buccal cortical layer, while in the femur there is the medullary canal. Both cancellous bone and medullary canal possess osteogenic cells. Patients requiring extensive tumor surgery after diagnosis of cancer of the floor of the mouth often have a history of extensive smoking, which limits the regenerative capacities of the oral mucosa and the bone tissue, since smoking impairs capillary function. In addition, with respect to osteosynthesis with mandibular segmental defects the elastic deformation of the mandible during chewing needs to be taken into account, this is, why it is important to use animal models (such as a mini-pig model) in which the same advanced osteosynthesis plates can be used that are used in patients, in order to achieve biomechanical competence and avoid failure of the defect model during the animal study (Fig. S1).
